# Quantifying the spatial spread of dengue in a non-endemic Brazilian metropolis via transmission chain reconstruction

**DOI:** 10.1038/s41467-018-05230-4

**Published:** 2018-07-19

**Authors:** Giorgio Guzzetta, Cecilia A. Marques-Toledo, Roberto Rosà, Mauro Teixeira, Stefano Merler

**Affiliations:** 1 Center for Information Technology, Bruno Kessler Foundation, via Sommarive 18, Trento, I-38123 Italy; 2 Epilab-JRU, FEM-FBK Joint Research Unit, Trento, I-38100 Italy; 3 Departamento de Bioquimica e Imunologia do Instituto de Ciencias Biologicas, Universidade Federal de Minas Gerais, Av. Antônio Carlos, 6627-Pampulha, Belo Horizonte, 31270-901 Minas Gerais Brazil; 4 Dipartimento di Biodiversità ed Ecologia Molecolare, Centro Ricerca e Innovazione, Fondazione Edmund Mach, via E. Mach 1, San Michele all’Adige (Trento), I-38010 Italy

## Abstract

The ongoing geographical expansion of dengue is inducing an epidemiological transition in many previously transmission-free urban areas, which are now prone to annual epidemics. To analyze the spatiotemporal dynamics of dengue in these settings, we reconstruct transmission chains in Porto Alegre, Brazil, by applying a Bayesian inference model to geo-located dengue cases from 2013 to 2016. We found that transmission clusters expand by linearly increasing their diameter with time, at an average rate of about 600 m month^−1^. The majority (70.4%, 95% CI: 58.2–79.8%) of individual transmission events occur within a distance of 500 m. Cluster diameter, duration, and epidemic size are proportionally smaller when control interventions were more timely and intense. The results suggest that a large proportion of cases are transmitted via short-distance human movement (<1 km) and a limited contribution of long distance commuting within the city. These results can assist the design of control policies, including insecticide spraying and strategies for active case finding.

## Abstract (web summary)

There is increasing urgency to understand the spatiotemporal dynamics of dengue in non-endemic regions. Here, the authors reconstruct likely dengue transmission chains in the city of Porto Alegre based on geo-located cases only, and find that most transmission events occur over short-distances.

## Introduction

Dengue is a mosquito-borne infection that causes symptomatic disease in 60 to 100 million persons per year and 14,000 to 20,000 annual deaths^1,2^, with a global health cost estimated at almost 9 billion dollars per year^2^. In Southern America, dengue and other arboviruses have strongly increased their circulation since the late 1990s, after the failure of continental control efforts that had come close to eliminate the vector mosquito in the previous decades^3^. Dengue transmission dynamics are determined by a complex interplay between environmental and climatic factors, abundance and competence of vector species, human density and behavior, ecological interactions among viral strains, and profiles of immunity in the population. Several studies have demonstrated the highly focal spatiotemporal dynamics of dengue in hyperendemic settings of South-East Asia, using genetic data from viral samples to infer phylogenetic trees^4–9^. These approaches have shown that, even when infection is widespread, most transmission events are locally clustered^8^, and further foci are occasionally exported to different areas via commuting and long-distance travels.

A more fine-grained characterization of the spatiotemporal spread of dengue can be obtained by the reconstruction of transmission chains, i.e., the sequence of who infected whom. Transmission chains can be identified directly through information collected within intensive field investigations (e.g., contact tracing)^10–14^. More often, transmission chains are inferred probabilistically by defining a likelihood for each epidemiological link dependent on the assumed spatial, temporal, and genetic relatedness among infections. The likelihood is defined by models that encode assumptions specific to the mechanism of transmission and the type of available data. In particular, a few studies are based on spatial and temporal data alone^12,15,16^, while, where available, genetic data have been used together with spatial and/or temporal data by combining epidemiological and evolutionary models (e.g.,^17–20^, reviewed in^21,22^). Inference is generally obtained via Markov Chain Monte Carlo (MCMC) exploration of the parameter space or, more rarely, by likelihood maximization^12^. To the best of our knowledge, only one study^14^ attempted the reconstruction of transmission chains for dengue, using contact tracing data on possible out-of-home exposure sites and a priori assumptions on the spatial and temporal distance between an infector and its infectees.

Here, we used a spatio-temporal transmission model based on MCMC parameter estimation to infer likely transmission chains in Porto Alegre, Brazil, from spatio-temporal notification data. In this way, we were able to provide critical quantitative information on the distance between infectors and infectees, and on the number of cases, diameter, and diffusion speed of transmission clusters (i.e., the set of secondary cases directly and indirectly caused by a single imported case). This information is crucial to design interventions.

## Results

### Transmission chains and reproduction numbers

**Fig. 1:**
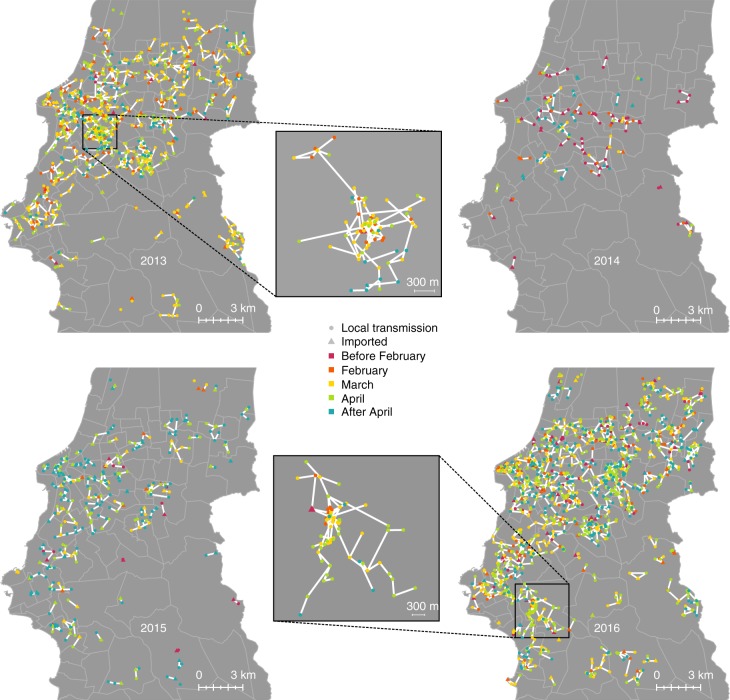
Spatio-temporal transmission chains. Maximum likelihood transmission chains occurring in Porto Alegre over the four years of study, with a detail on the two largest clusters. Triangles represent imported cases and circles represent locally transmitted cases. Different colors are used to show the time of symptom onset. White lines indicate a transmission link between cases; light gray boundaries indicate borders between administrative neighborhoods

**Fig. 2:**
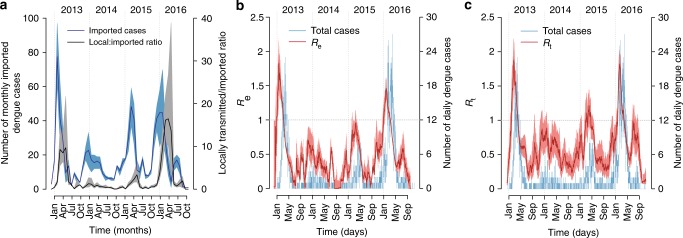
Epidemic course and reproduction numbers. **a** Estimated mean number (and 95% CI) of imported cases over time (blue line and shaded area, scale on the left) and mean ratio (and 95% CI) of the number of locally transmitted cases to the number of imported cases over time (black line and shaded area, scale on the right). **b** Effective reproduction number *R*_e_ (mean and 95% CI) over time, compared with daily cases. **c** Instantaneous reproduction number *R*_t_ (mean and 95% CI), computed from the renewal equation

Figure 1 shows a spatio-temporal representation of the maximum-likelihood transmission chains occurring in Porto Alegre over the four years of study with a detail on the two largest clusters, in 2013 (92 cases) and 2016 (116 cases). In these two years, intense local transmission of dengue occurred over the whole city, while transmission was mostly sporadic in 2014 and 2015. In particular, during 2014 there was a reduced inflow of imported cases, reflecting the low dengue incidence in Brazil in the same year (less than 400 cases per 100,000, about half that of 2013, 2015, and 2016^23^). In 2015, instead, the dengue season in Brazil started later than other years^24^, so that importations in Porto Alegre remained rare until the months of March and April, when the mosquito season was already declining. According to both epidemiological data and model classification, imported dengue cases peaked about one month before autochthonous cases, i.e., between January and March (Fig. 2a). This pattern possibly reflects the return of travelers from Christmas and summer holidays and Carnival celebrations in endemic regions of Brazil.

The model estimated an average generation time (i.e., the time between successive infections in a transmission chain) of 17.3 days (95% CI: 16.7–18.0 days), in excellent agreement with previous estimates for dengue, ranging between 15 and 19 days^25^. We found that the effective reproduction number *R*_e_ (i.e., the mean number of secondary cases generated by a primary infector, see Methods) exceeded the epidemic threshold only between January and mid-March in 2013 and 2016, with an average peak during the month of highest transmission of 1.57 (95% CI: 1.25–1.92) in 2013 and 1.34 (95% CI: 1.19–1.49) in 2016 (Fig. 2b). A strikingly similar curve can be obtained by computing the instantaneous reproduction number *R*_t_ (see Methods), with average values of *R*_t_ in the month of highest transmission equal to 1.61 (95%CI: 1.35–1.91) in 2013 and 1.66 (95%CI: 1.47–1.88) in 2016 (Fig. 2c). These values are consistent with estimates obtained for dengue epidemics in other Brazilian cities^9^.

### Characteristics of transmission clusters

**Fig. 3:**
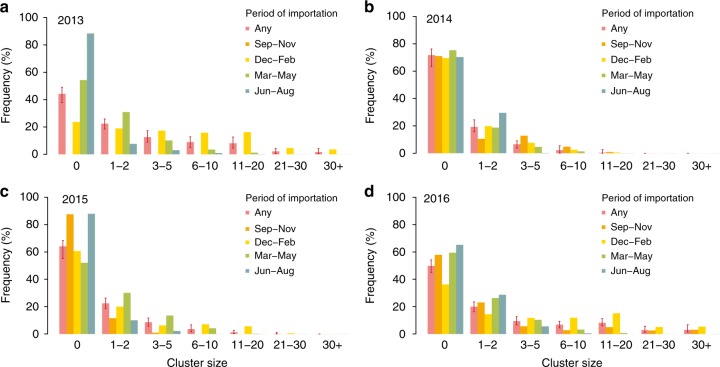
Cluster size distribution by year and period of importation. Distribution of cluster size by year (and 95%CI) as resulting from the analysis of all transmission clusters, or by considering clusters seeded in different time periods. Index cases are not considered in the computation of the cluster size. Different panels (**a**-**d**) refer to different epidemiological years

About 50% of imported cases did not result in symptomatic secondary cases during 2013 and 2016, compared to about 60% in 2015 and 75% in 2014 (Fig. 3). Transmission was sporadic for most clusters, but 13% of them in 2013 and 16% in 2016 were larger than 10 cases; these made up 57% and 69% of all transmitted cases in the respective year and persisted for a median duration of 113 days (95% CI: 56–196 days). The figure also shows that the largest clusters were systematically seeded in the period of highest transmissibility (December to February).

**Fig. 4:**
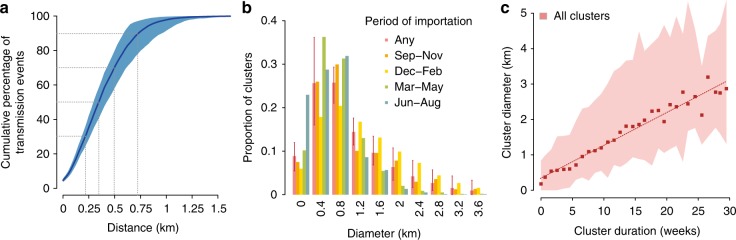
Focal transmission of dengue. **a** Estimated cumulative proportion (and 95%CI) of transmission events as a function of distance. Dashed lines highlight transmission distances at 30, 50, 70, and 90-percentiles of transmission events. **b** Distribution of the cluster diameter (and 95%CI) as resulting from the analysis of all transmission clusters, or by considering clusters seeded in different time periods. **c** Relation between cluster diameter and duration as resulting from the analysis of all transmission clusters; points: average diameter; shaded area: 95% CI; dashed line: linear regression

The average distance between the residence of infectees and that of infectors was estimated at 382 m (95% CI: 316–480 m), with over 97% of transmissions occurring within 1 km (Fig. 4a). Considering that the flight range of *Aedes aegypti* is around 100 m^26^, this result suggests a significant impact for the spread of disease of short-distance human movements, for example by walking through common areas such as parks^14^ or via house-to-house visits^13^. It must be noted that the model might have systematically missed long-distance, within-city transmission events (e.g., due to commuting-related mobility). Such events are expected to produce a number of geographically isolated cases in neighborhoods without active dengue transmission, which the model would misclassify as imported due to the absence of suitable potential infectors in the neighborhood. Comparing cases labeled as imported by the model with corresponding epidemiological evidence from lab-confirmed cases, we show that such a misclassification occurred in less than 5% of known autochthonous cases (see Supplementary Discussion), suggesting that longer-distance movements might be responsible only for a limited proportion of dengue transmission events within the city.

The majority of clusters (60%) did not spread beyond an area of diameter 1 km, with very few (3.2% overall) expanding beyond 3 km (Fig. 4b). Clusters seeded between December and February tended to spread significantly more than those seeded by cases imported earlier or later in the season. The geographical expansion of the cluster diameter over time showed a stable linear trend (Fig. 4c). Clusters reached a diameter of 1 km on average after about 7 weeks since importations, with a maximum of 2.3 km in the same period.

**Fig. 5:**
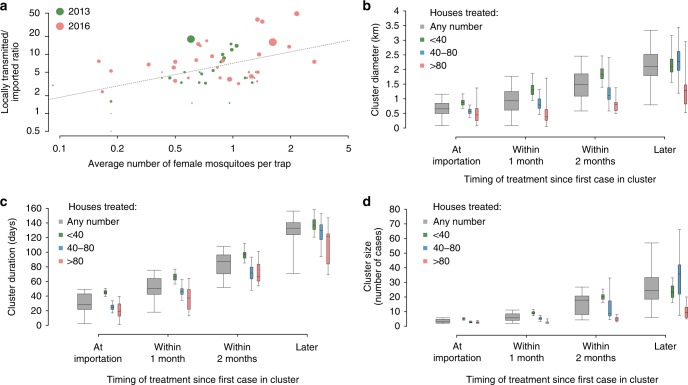
Effect of mosquito abundance and treatments. **a** Relationship between transmission intensity, represented by the ratio of local to imported cases, and the neighborhood’s mosquito infestation level (log-log scale). Circles: neighborhoods for which the information on mosquito abundance was available (size is proportional to the total number of cases). Dashed gray line: linear regression on the log-transformed variables (*p*-value = 0.0001; *R*^2^ = 0.26). **b**–**d** Impact of timing and intensity of vector-control on cluster diameter (**b**), duration (**c**) and size (**d**). Center: mean; bounds of box: interquartile range; whiskers: 95%CI

We found a robust relationship between the model-estimated transmission intensity and the corresponding mosquito infestation in neighborhoods for which this information was available (29 in 2013 and 40 in 2016, Fig. 5a; in 2014 and 2015 there were too few autochthonous cases to obtain stable neighborhood-specific estimates). Specifically, the ratio of locally transmitted to imported cases, which is an indirect measure of the transmission potential, increased by 57% (95%CI: 25–97%) for every doubling in the number of mosquitoes collected per trap. Cluster characteristics were found to vary significantly with respect to vector control interventions implemented within the area and time of the cluster (Fig. 5b–d). More specifically, the diameter, duration and size of a cluster were proportionally smaller when interventions were more intense (higher number of households in the treated area) and more timely (shorter delay between the symptom onset of the cluster’s index case and day of adulticide spraying). For example, the average diameter was from 40% to 70% smaller in clusters where treatments involved more than 80 households, compared to those where less than 40 households were treated; a similar difference was found for clusters with treatment administered on the date of importation compared to those implemented more than 2 months later.

### Sensitivity analyses

To test the robustness of our conclusions with respect to potential underreporting in our dataset, we reproduced our analysis on (i) the subset of laboratory confirmed cases, which represent about 20% of all notifications; (ii) a random subsample of 60% of all notifications. In both cases, we found similar generation times (average 17.7 and 16.9 days, respectively) and an even more local spread (average transmission distance 223 and 346 m, respectively). Clusters were smaller (about 80% had a final diameter of less than 1 km in both experiments) and had a slower geographical expansion (see Supplementary Discussion).

Furthermore, using synthetic data from a dynamic transmission model we were able to reconstruct epidemiological parameters and transmission chains with good accuracy: provided that the reporting rate remained above or equal to 50%, the kernel parameter, average transmission distances and generation times were identified with a relative error of less than 15%; furthermore, over 60% of transmission links were correctly identified by the model in at least one of the reconstructed chains. For lower reporting rates the model systematically overestimated the transmission distance and the number of identified links dropped to about 40% (see Supplementary Discussion). These analyses showed that completeness of infection data is not necessary for inferring the spatiotemporal structure of transmission chains, reducing potential biases due to underreporting.

Finally, we fitted a model with an alternative function regulating the distance-dependent probability of transmission. In particular, we used a radiation kernel^27^ to represent human commuting in place of the exponential kernel adopted in the main analysis. Briefly, a much larger mean transmission distance was estimated (1174 m, 95%CI: 1119–1244), cluster diameter expanded up to 10 km, thus covering large parts of the city, and the diffusion speed was estimated at roughly 2 km month^−1^ (see Supplementary Discussion). The likelihood associated to this model, however, was much lower than that obtained by adopting the exponential kernel (see Supplementary Discussion).

## Discussion

Using a Bayesian inference model calibrated to geo-located surveillance data, we demonstrated the highly focal diffusion of dengue in Porto Alegre, Brazil, a non-endemic metropolitan city undergoing an epidemiological transition from a transmission-free setting to one with frequent epidemics. The majority of transmission events occurred in individuals residing within 400 m from an infectious case, and only exceptionally beyond 1 km. Clusters of transmission expanded locally with a diameter growing slowly, by approximately 1 km every 7 weeks. Thus, because of the relatively short seasonal transmission of dengue in Porto Alegre, the majority of clusters did not extend beyond 1 km. These results suggest a strong contribution to local cases of walking-distance human movements, and the less frequent occurrence of transmission across longer ranges (e.g., due to urban commuting); the latter, however, may seed the infection in new areas that may become further foci of local dengue diffusion. The high frequency of importations confirms the importance of travel to and from endemic areas in sustaining the occurrence of local outbreaks in non-endemic settings.

No evidence of superspreading was found at the individual level, however a small number of uncontrolled clusters (about 15% of the total) were estimated to be responsible for a large proportion (55–70%) of all cases in years with intensive transmission (2013 and 2016). We estimated that timely administration of adulticides taking place in public areas at the time of importation of an index case was capable of reducing the cluster size by a factor of 3 to 10 compared to late interventions. Interventions covering a large number of houses (either because of higher housing densities or because larger areas were treated) also resulted in improved infection control. Vector control, however, did not influence the qualitative dynamics of a cluster’s spatiotemporal spread in terms of transmission distances and cluster diffusion rates.

Porto Alegre is a Brazilian metropolis of over 1,400,000 inhabitants, where dengue infection has started to be locally transmitted only since 2010 and the first large epidemics occurred in 2013. The presence of an intensive surveillance system provided a unique chance to investigate the spatio-temporal transmission dynamics using notification data that are representative of almost the full history of infection throughout the whole city. The humid subtropical climate (Koppen Geiger classification: Cfa^28^), with colder temperatures compared to the rest of Brazil (largely tropical), allowed for lower mosquito abundances and transmission probabilities^29^. Therefore, the yearly incidence of notified cases in Porto Alegre in 2013–2016 (between 15 and 150 cases per 100,000 inhabitants) was closer to the national average of Argentina than to the Brazilian one (300–700 per 100,000)^23,30^. These low-incidence conditions, combined with the recent emergence of local transmission, greatly simplify the transmission dynamics by avoiding issues related to pre-existing immunity levels and the ecological competition due to serotype cross-immunity.

The dengue surveillance system of Porto Alegre was designed for an intensive monitoring of a large metropolitan area; at this scale, passive surveillance (augmented by epidemiological investigations to determine importations from outside the city) is the most practical option, given that comprehensive active surveillance or molecular epidemiological analyses would be logistically and economically burdensome. In general, surveillance data may suffer from underreporting due to asymptomatic infections and unrecognized symptomatic cases. In Brazil, the proportion of clinically inapparent dengue is consistently estimated at around 40%^31–34^, much lower than the world average of 75%^1^. In Porto Alegre, underreporting rates may be even lower, since the frequency of subclinical cases is positively correlated with the incidence of disease in previous years^34^, possibly reflecting the role of pre-existing immunity in reducing the severity of symptoms. In addition, asymptomatic cases play a role in transmission chains only if they are able to further transmit the infection. Recent studies have suggested that some asymptomatic and pre-symptomatic children might be able to infect mosquitoes^35^; however, evidence on how often this translates into actual transmission to humans is lacking, and there is no data on transmission from asymptomatic adults (children below 15 years old represent only 7% of notified cases in Porto Alegre). Overall, the transmissibility of asymptomatic individuals is generally assumed to be much lower than that of symptomatic patients^36^. These considerations reduce the risk of biases associated to underreporting in notification data. Our analyses on synthetic data sets (see Supplementary Discussion) showed a robust reconstruction of the spatiotemporal dynamics even when considering underreporting rates of up to 50%. However, for higher underreporting, the model systematically overestimated the average transmission distance; this suggests that cluster size might be larger than estimated and that the spatiotemporal spread of dengue might be even more local if asymptomatic transmission and missed notifications play a significant role in Porto Alegre. However, our conclusions remained consistent even when considering subsets of the notified data in Porto Alegre with undersampling rates of up to 40% or when considering only confirmed cases (see Supplementary Discussion).

The features of our inferred transmission chains are consistent with recent results on clusters reconstructed from molecular clock analyses on dengue epidemics in Thailand^8^. In particular, we confirm that the number of clusters in an area increases with population density and that the proportion of cases belonging to the same cluster of a given case decreases rapidly with distance from the case (see Supplementary Discussion). Our results are also in qualitative agreement with findings from a large dengue epidemic in Cairns, Australia^14^, where 95% of transmission events were localized within the city (urban radius of 2.5 km), although human commuting was found to have a major role in seeding foci of infection to peri-urban towns. Our analyses show that a model explicitly accounting for commuting within the city is less able to explain the observed spatio-temporal dynamics of dengue in Porto Alegre (see Supplementary Discussion).

Viral genetic data or contact tracing information can overcome some of the downsides of passive surveillance data and strengthen the inference process. Nonetheless, in this study we were able to robustly reconstruct transmission chains using only the date of onset and geographical positions of recorded cases. This inference allowed for the first time a detailed quantitative characterization of the spatiotemporal spread of dengue clusters in a large, non-endemic metropolis, over multiple years. The ongoing geographic expansion of dengue due to the intensification of international travels^37^, urbanization^38^ and to climatic adaptation of mosquitoes and viruses^39^, puts a growing number of cities worldwide (e.g., in USA, Southern Europe, Australia or subtropical South America) in an epidemiological transition similar to the one currently faced by Porto Alegre. Results from this study may be crucial for assisting the appropriate design of vector-control interventions aimed at preventing or limiting disease spread in these areas and help public health policy makers to enhance disease surveillance and plan active case finding strategies. Finally, reported results can be used to support the research related to optimal vaccine^36^ and drug deployment^40^.

## Methods

### Geographical setting

Porto Alegre (30°01′40″S, 51°13′43″W) is the main city of Rio Grande do Sul, the southernmost state of Brazil. The city has an area of 496.68 km^2^, an estimated population of 1,409,351 inhabitants, and a high human development index of 0.81^41^. The climate is classified as subtropical humid (Cfa)^28^. In Porto Alegre, the first record of an imported dengue case occurred in 2002^42^, but first local transmission was confirmed only in 2010^43^. The city of Porto Alegre has experienced several episodes of dengue introduction in the last few years and has developed an integrated surveillance and prevention protocol that includes entomological, virological, and epidemiological components^30^. Even though the incidence of dengue cases in the southern region of Brazil has increased in the last years, it is much lower than other regions of the country^44^, due to the temperate climate^28^. In a seroprevalence survey conducted in the city from July to September 2015 (unpublished data), 422 serum samples of blood donors, homogeneously distributed across the city area, were tested for anti-Dengue IgG antibodies, and only 1 was found positive (95% confidence intervals (CI) estimates for the seroprevalence: 0.01–1.3%).

### Data

Dengue notification data were obtained from the Brazilian Ministry of Health. A fraction of suspected cases was confirmed by immunological tests (ELISA), and in such case epidemiological investigations were performed, consisting in the administration of a questionnaire aimed at ascertaining whether the case was imported or locally transmitted (the form is reported in Portuguese in the Supplementary Methods). Cases were considered imported if they had a travel history to dengue endemic areas in the 10 days preceding symptom onset. Cases were considered locally transmitted based on the presence of other dengue cases in the vicinity of the patient’s residence, and on possible identification of direct links with social contacts who were recently diagnosed with dengue. Overall, 3413 cases of dengue were recorded in Porto Alegre between December 2012 and October 2016, of which 646 were lab confirmed (Table 1). The date of symptom onset and the geographical coordinates of the patients’ residence were available for all cases. Dengue type was known for only 30 cases; among these, 29 belonged to type 1. However, the negligible pre-existing population immunity greatly simplifies the complex ecological interplay among serotypes, allowing to analyze the data independently of dengue type.

**Table 1:** Characteristics of observed dengue cases

	2013	2014	2015	2016	Overall
Total number of cases	1057	264	485	1607	3413
Confirmed with known origin (%)	210 (19.9)	16 (6.1)	72 (14.8)	348 (21.7)	646 (18.9)
Known imported (% of confirmed)	71 (33.8)	11 (68.8)	54 (75.0)	56 (16.1)	192 (29.7)
Known local (% of confirmed)	139 (66.2)	5 (31.2)	18 (25.0)	292 (83.9)	454 (70.3)

Epidemiological years are assumed to run between November 1st of the preceding year to October 31st of the same year

Mosquito data were based on the collection of adult *Ae. aegypti* mosquitoes by the local monitoring system^45,46^, using the sticky trap MosquiTRAP (Ecovec LTDA, Brazil). The surveillance system has 935 sticky traps distributed in an area covering 44% of the city, at a distance of 250 m between each other, as described previously^45^. The traps were inspected weekly. The entomological index provided by the system is the Index of Mean Female *Ae. aegypti* (IMFA), given by the total number of captured *Ae. aegypti* females, divided by the number of inspected traps.

Ultra-low volume adulticide treatment was started in public spaces within a radius of 50 or 200 m around the residence of a confirmed case and the number of houses in the treated area was recorded. Treatments were started for 75% of the lab-confirmed imported cases and 60% of lab-confirmed autochthonous cases, as well as for 106 non-confirmed cases (4%).

### Transmission model and inference

Following the classical SEIR (Susceptible-Exposed-Infected-Removed) infection model for dengue in humans^47,48^, we assume that, at any time *t*, susceptible individuals are exposed to a force of infection

$$\lambda _j\left( t \right) = \mathop {\sum}\limits_{i \in N(t)} {\beta K(d_{ji};\eta )\Gamma (t - E_i;a,b)}$$

where *N(t)* is the set of individuals who have been infected before time *t* and *β* is the disease transmission rate. $K\left( {d_{ji};\eta } \right) = \eta e^{ - \eta d_{ji}}$ is the spatial kernel regulating the probability of transmission at a distance *d*_*ji*_ between individuals *j* and *i*. *E*_*i*_ is the time of infection of individual *i* and Γ(*t*; *a*, *b*) is the gamma-distributed generation time (i.e., the time between successive infections in a transmission chain), with shape parameter *a* and rate parameter *b*. The generation time accounts for the length of incubation period in both humans and mosquitoes (intrinsic and extrinsic incubation periods^49^), duration of human infectiousness and lifespan of mosquitoes; we assume the generation time to be proportional to the infectiousness profile. In the time interval [*t*, *t*+Δ*t*], susceptible individuals get infected with probability $p_j(t) = 1 - e^{\lambda _j(t){\mathrm{\Delta }}t}$, and have a constant probability *α* to be a dengue case imported in the study area. Putative transmission chains were defined by selecting, for each case *j*, a candidate infector *i* on the basis of the force of infection exerted by *i* on *j* at time *E*_*j*_; the likelihood that a case was imported from outside the city rather than locally infected was also accounted for with a constant probability^15^. The likelihood of the full transmission chain was then evaluated on the basis of two terms for each case: one accounting for the probability of being infected at time *E*_*j*_, and one accounting for the probability of remaining susceptible until time *E*_*j*_. Model parameters were estimated using an MCMC algorithm with uninformative priors (uniform distributions) and random-walk Metropolis–Hastings sampling with reversible jumps from normal distributions (see Supplementary Methods for full details); transmission chains defined by parameter sets accepted during the MCMC were considered for the analysis of clusters.

### Cluster and geographical analysis

We defined a transmission cluster as the set of secondary cases directly and indirectly caused by a single imported case. For each cluster, we evaluated its size (number of cases), diameter (maximum distance between any two cases), and duration (time elapsed between the symptom onset of the first and last case). Results were additionally disaggregated over the 81 neighborhoods of Porto Alegre in which dengue cases (probable or confirmed) were registered. We defined a measure of transmission intensity as the ratio of local to imported cases in a given year and neighborhood, and an index of mosquito infestation, computed as the average number of female mosquitoes captured^45^ in traps within 150 m from a case and within 4 weeks before symptom onset.

### Reproduction numbers

The basic reproduction number *R*_0_ is defined as the average number of secondary infections caused by a typical primary infection in a fully susceptible population. When *R*_0_ is larger than *1*, the epidemic may spread in the population with intensity proportional to *R*_0_. For mosquito-borne diseases, the reproduction number may vary over time, depending on the local abundance of mosquitoes. Thus, we computed the instantaneous reproduction number *R*_*t*_ by approximating the number of locally transmitted cases over time with the renewal equation^50^ (see Supplementary Methods). We also computed the effective reproduction number *R*_e_ over time from the reconstructed transmission chains as the average number of secondary cases caused by individuals who had symptom onset during a moving temporal window of one month. While *R*_e_ depends on all the characteristics of the reconstructed transmission chains, *R*_t_ depends only on estimates of the generation time and information on the number of imported cases over time.

### Code availability

C code is available from the corresponding author upon request.

### Data availability

The data that support the findings of this study are available on request from the corresponding author S.M. Individual patient data are not publicly available to protect research participant privacy/consent.

## Electronic supplementary material

Supplementary Information
