# Supplementary material for: Quantifying the spatial spread of dengue in a non-endemic Brazilian metropolis via transmission chain reconstruction
Source: Nat Commun. 2018 Jul 19;9:2837. doi: 10.1038/s41467-018-05230-4 (PMC6053439; doi:10.1038/s41467-018-05230-4)
Supplement: Supplementary file 1 — Supplementary Information [file 41467_2018_5230_MOESM1_ESM.pdf]

Quantifying the spatial spread of dengue in a non-endemic Brazilian metropolis via transmission chains reconstruction

Guzzetta et al.

Supplementary Information

Supplementary Methods

Here we describe the transmission model used for transmission chains reconstruction. We assume that, at any time t, susceptible individuals are exposed to a force of infection

$$\lambda_j(t) = \sum_{i \in N(t)} \lambda_{ij}(t) = \sum_{i \in N(t)} \beta K(d_{ji}; \eta) \Gamma(t - E_i; a, b)$$

where $N(t)$ is the set of individuals who have been infected before time t and β is the disease transmission rate. $K(d_{ji}; \eta) = \eta e^{-\eta d_{ji}}$ is the spatial kernel regulating the probability of transmission at a distance d_{ji} between individuals j and i. The kernel's "characteristic distance" is defined as $1/\eta$. E_i is the time of infection of individual i and $\Gamma(t;\ a, b)$ is the gamma-distributed generation time, with shape parameter a and rate parameter b. The generation time accounts for the length of incubation period in both humans and mosquitoes (intrinsic and extrinsic incubation periods), duration of human infectiousness and lifespan of mosquitoes. In the time interval $[t, t + \Delta t]$, susceptible individuals get infected with probability $p_j(t) = 1 - e^{\lambda_j(t)\,\Delta t}$, and have a constant probability α to be a dengue case imported in the study area.

Here we describe the parameter estimation procedures. For each set of free parameters $\theta = \{\alpha, \beta, \eta, a, b\}$, we reconstruct the transmission chain by sampling, for each case j, among all infected individuals i such that $E_j - E_i$ is in the 99% percentile of $\Gamma(t; a, b)$, a likely infector k_j with probability proportional to $\beta K(d_{ji}; \eta)$; the case that j was imported was considered by including the probability of importation α as the transmission probability of an additional candidate infector [1]. The likelihood of the transmission chain given parameter set θ will be given by:

$$L(\theta) = \prod_j P(j, k_j)\ Q(E_j)$$

where

$$P(j, k_j) = \begin{cases} \alpha & \text{if } j \text{ is imported} \\ \lambda_{jk_j}(\mathrm{E}_j) & \text{if } j \text{ has been infected by } k_j \end{cases};$$

λ_{ik_j} represents the likelihood of having been infected by individual k_j, while α represents the likelihood of importation; $Q(E_j)$ is the likelihood that j has not been infected nor imported before time E_j, namely:

$$Q(E_j) = \exp\left(-\int_0^{E_j} \alpha + \lambda_j(t)\, dt\right)$$

Time 0 was set at November 1st, 2013, i.e. the first day of the first epidemiological year in the dataset.

We estimated unknown parameters defined by vector $\boldsymbol{\theta} = \{\alpha, \beta, \eta, a, b\}$ with a Markov Chain Monte Carlo procedure based on uninformative priors for all parameters (Supplementary Table 1) and random-walk Metropolis-Hastings sampling with reversible jumps from normal distributions. The starting point for parameter values was selected by running 10,000 runs with values sampled from the prior uniform distributions (Supplementary Table 1) and accepted with a maximum likelihood criterion. This procedure was used to choose an initial parameter set with likelihood numerically strictly greater than 0. A new parameter set $\boldsymbol{\theta}_{\mathbf{new}}$ was proposed by adding to the last accepted parameter set $\boldsymbol{\theta}_{\mathbf{old}}$ a vector of samples from normal distributions with mean zero and standard deviations $\boldsymbol{\sigma}$ (Supplementary Table 1); for

each parameter $\vartheta \in \boldsymbol{\theta}$ a new value was proposed as $\vartheta_{new} = \vartheta_{old} + N(0, \sigma_\vartheta^2)$. $\boldsymbol{\theta_{new}}$ was accepted depending on the likelihood of its reconstructed chain with probability equal to:

$$\pi = \min\left(1, \frac{L(\boldsymbol{\theta}_{\mathbf{new}})}{L(\boldsymbol{\theta}_{\mathbf{old}})}\right)$$

In our datasets, the time of symptom onset and the geographic location of all considered cases are known, but not the times of infection $\boldsymbol{E} = \{E_j\}_j$. The above described procedure could be used to estimate model parameters only if infection times $\boldsymbol{E}$ were known. A standard approach is to consider infection times $\boldsymbol{E}$ as nuisance parameters to be estimated together with model parameters (for instance by using an independence sampler for sampling proposal infection times) and use data augmentation techniques to incorporate these missing data [1-3]. In principle, all infection times $\boldsymbol{E}$ should be updated at each MCMC step. Previous studies have suggested a two-stepped optimization of nuisance parameters and model parameters within the Markov Chains [1,2]; in some cases, only a subset of values of $\boldsymbol{E}$ are updated at each optimization step [3]. Here, we propose a different approach that is more scalable with larger sample sizes as it does not require to control a large number of nuisance parameters. We created 2,000 augmented data sets where, for each case j, we randomly sampled an incubation period τ_j between 3 and 10 days from a uniform distribution [4] and imputed the value of E_j by subtracting the sampled τ_j from the observed time of symptoms onset. Model calibration was run over each augmented dataset, using 100,000 MCMC iterations, of which only the final 10,000 were kept to ensure convergence of the algorithm. Results from the 2,000 Markov Chains were pooled together, obtaining 20,000,000 total samples for the posterior distributions. Finally, a transmission chain was considered in the analysis for each accepted parameter set. The great computational advantage of the proposed method is that the 2,000 Markov Chain can be computed in parallel. As shown in Supplementary Figure 1, the posterior distributions of model parameters stabilize when pooling together more than 300 randomly sampled Markov Chains. This result shows that 2,000 Markov Chains are sufficient to account for uncertainty on infection times $\boldsymbol{E}$ in estimating the posterior distributions of model parameters. The proposed method is demonstrated on synthetic data sets (see below).

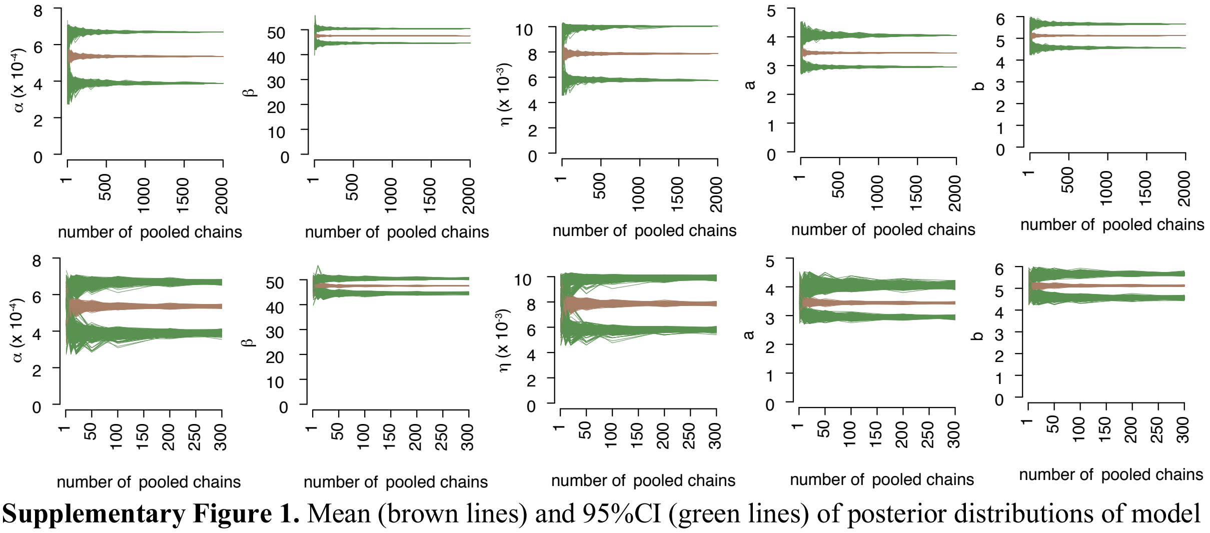

Supplementary Figure 1. Mean (brown lines) and 95%CI (green lines) of posterior distributions of model parameters as obtained by pooling together an increasing number of Markov Chains (the final 10,000 steps only were considered) randomly sampled among the 2,000 Markov Chains computed in parallel. The sampling procedure is repeated 1,000 times. Panels in the bottom row show the same results in the top row panels, but for a maximum number of pooled Markov Chains set to 300.

Parameter	Prior distribution	MCMC σ	Posterior distribution: mean (95%CI)	Unit
α	Uniform(0, 1)	10^{-5}	5.36 (3.87 – 6.68) 10^{-4}	days^{-1}
β	Uniform(0, 1000)	1	47.6 (44.6 - 50.4)	days^{-1}
η	Uniform(0, 1)	10^{-4}	7.86 (5.75 – 10.0) 10^{-3}	m^{-1}
a	Uniform(0, 100)	0.1	3.44 (2.95 – 4.05)	-
b	Uniform(0, 100)	0.1	5.12 (4.56 - 5.66)	-

Supplementary Table 1. Prior distributions, standard deviations of normal jumps and posterior distribution for model parameters

Supplementary Table 1 shows the posterior mean and 95% confidence intervals for all parameters, while Supplementary Figure 2 represents the resulting probability distribution functions for the generation time and the spatial kernel.

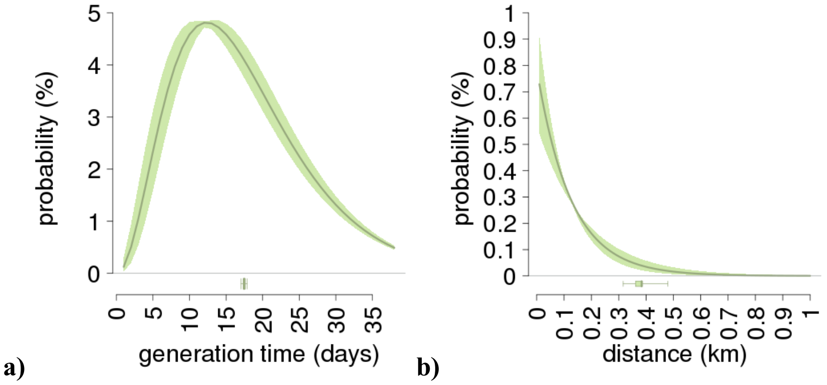

Supplementary Figure 2. a) Probability distribution of the generation time and **b)** spatial kernel, computed from the posterior distributions of parameters. Dark line: average; shaded area: 95% credible intervals. Boxplots below the zero line show for comparison the mean, inter-quantile range and 95% credible interval of the generation time and transmission distance in the reconstructed transmission chains.

Here we describe the procedures for computing reproduction numbers. To estimate the instantaneous reproduction number $R_t$we approximate the number of locally transmitted cases over time with the renewal equation:

$$\mathrm{C(t) - A(t) = Pois}\left(\mathrm{R_t}\sum_{\mathrm{s=1}}^{\mathrm{t}} \mathrm{C(t-s)\Gamma(s;a,b)}\right),$$

where *C(t)* is the total number of cases with symptom onset at time *t*, *A(t)* is the number of imported cases with symptom onset at time *t*, and Γ is the generation time distribution, with shape and scale parameters a and b sampled from the posterior distribution estimated by the model for the reconstruction of transmission chains. The likelihood is therefore:

$$L = \prod_{t\geq 1} p\left(C(t) - A(t), \mathrm{R_t}\sum_{\mathrm{s=1}}^{\mathrm{t}} \mathrm{C(t-s)\Gamma(s;a,b)}\right).$$

where *p(k,λ)* is the probability mass function of a Poisson distribution (i.e. the probability of observing *k* events if these events occur with a known rate *λ*). We estimated mean and 95% credible intervals of R_t by the same MCMC procedure described above. We run 100,000 MCMC iterations to identify the posterior distributions of R_t, assuming prior uniform distribution, namely Uniform(0,10), using a constant value of 0.1 for the standard deviations of the normal jumps, and we kept the last 10,000 iterations to ensure convergence of the

algorithm. We considered *A(t)* as the number of cases classified as imported in more than 50% of the reconstructed transmission chains.

We also computed the effective reproductive number R_e over time from the reconstructed transmission chains as the average number of secondary cases caused by individuals who had symptom onset during a moving window of one month. While R_e depends on all the characteristics of the reconstructed transmission chains, R_t depends only on estimates of the generation time and information on the number of imported cases over time.

Here we describe the generation of synthetic data used for validating the procedures for reconstructing the transmission chains. We tested our transmission chain reconstruction model on synthetic data obtained from the simulation of a stochastic transmission model with known structure and parameters. In the transmission model, susceptible individuals become exposed within a given time interval $[t, t+\Delta t]$ with a probability

$$p_i(t) = 1 - e^{\lambda_i(t)\,\Delta t},$$

where $\lambda_i(t)$ is a time-dependent force of infection:

$$\lambda_i(t) = \beta(t)\sum_{j=1}^{N} I_j K(d_{ij}, \eta)$$

- $\beta(t) = \beta_0|\sin(\frac{\pi t}{365})|$ is a time-varying transmission rate and *t* is the simulation day;
- *N* is the total population;
- I_j is 1 if the individual is infectious and 0 otherwise
- $K(d_{ij}, \eta) = \eta e^{-\eta d_{ij}}$ is a spatial kernel regulating the probability of transmission at a distance d_{ij} via parameter η. We term $1/\eta$ the "characteristic distance" of the kernel.

When an individual is exposed, his infector is chosen among the set of individuals who are infectious at that time with a probability proportional to $K(d_{ij}, \eta)$. The natural history of disease in individuals follows an SE^2I^2R model [5], where the total incubation time T_E of exposed individuals is given by the sum of two times T_{E1} and T_{E2}, each sampled from an exponential distribution with mean 7 days; similarly, the total infectious period T_I is given by the sum of two times T_{I1} and T_{I2}, each sampled from an exponential distribution with mean 3 days. In this way, T_E and T_I are approximated by two gamma distributions: $\Gamma_{T_E}(2{,}7)$ and $\Gamma_{T_I}(2{,}3)$ with means of 14 and 6 days respectively [4]. The theoretical average generation time in a homogeneous mixing SE^2I^2R model is $\bar{T}_g = \bar{T}_E + \frac{3}{4}\bar{T}_I$ [5], so that $\bar{T}_g = 18.5$ days. However, due to local saturation effects in the spatial model, the empirical generation time is slightly lower, with an average of about 17 days (Supplementary Table 2).

Parameter	Value	Unit
β_0	3.19	$days^{-1}$
η	$5.3\ 10^{-3}$	m^{-1}
$\bar{T}_E$	14	days
$\bar{T}_I$	6	days

Supplementary Table 2. Model parameters for the generation of synthetic datasets.

Synthetic datasets were built by running the transmission model over a square region of side length Q by seeding 200 imported cases at times sampled with uniform probability in the interval [1, 365] and a random location with uniform probability over space. The model was run until there were no more exposed or infectious individuals. Smaller values of Q result in

more densely distributed clusters, and therefore in a higher difficulty for the reconstruction model to discriminate among separate clusters occurring over the same area. A number of datasets were built by simulating the model with different values of Q (between 4 and 40km); the resulting number of total cases ranged between about 1400 and 1800, i.e. in the range of suspected and confirmed dengue cases in Porto Alegre in the year with highest incidence (i.e., 2016). The number of imported cases in the synthetic datasets (200) is also within the expected range of imported dengue cases for Porto Alegre based on both confirmed cases and on the model classification of suspected cases (see main text). A simulated synthetic data set is shown in Supplementary Figure 3. The reconstruction model was calibrated to each dataset using the same MCMC procedure reported above and in in the main text.

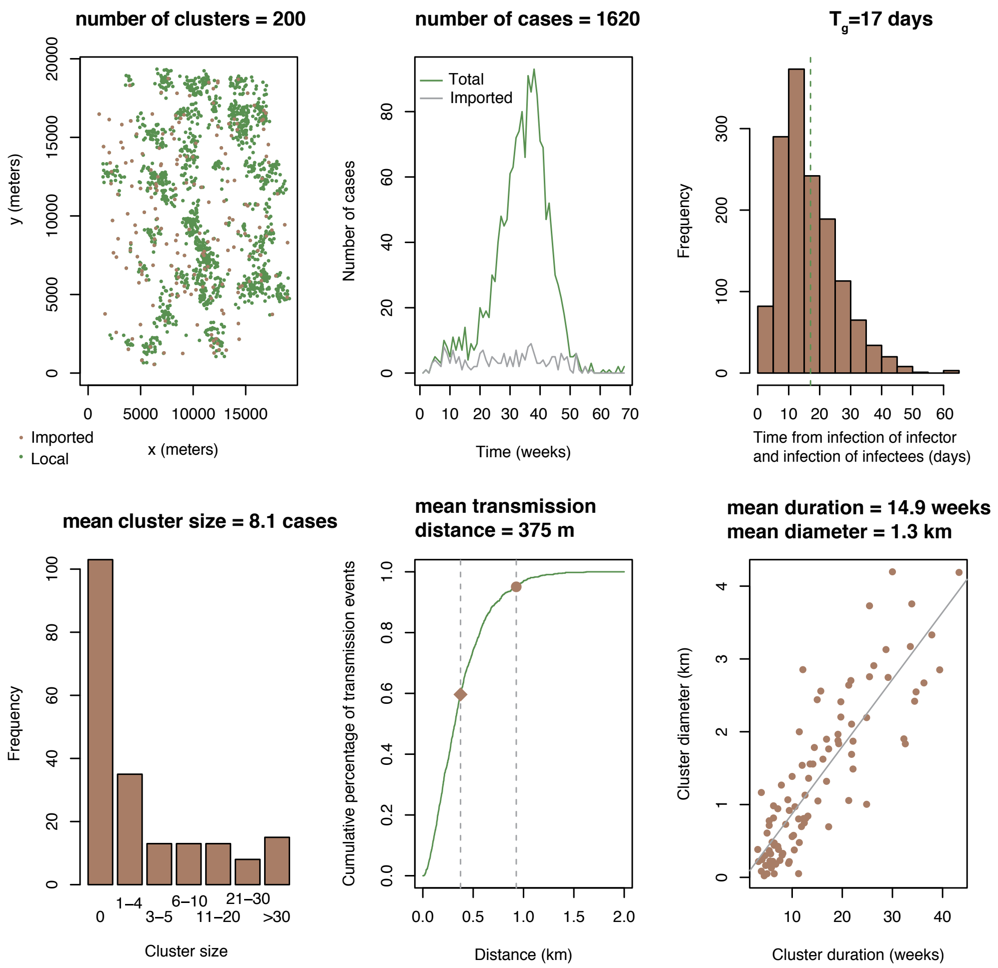

Supplementary Figure 3. Characteristics of the synthetic data sets for Q=20km.

In the next page, we provide the form adopted for epidemiological investigations (in Portuguese).

SINAN

República Federativa do Brasil
Ministério da Saúde

SISTEMA DE INFORMAÇÃO DE AGRAVOS DE NOTIFICAÇÃO

FICHA DE INVESTIGAÇÃO DENGUE E FEBRE DE CHIKUNGUNYA Nº

Caso suspeito de dengue: pessoa que viva ou tenha viajado nos últimos 14 dias para área onde esteja ocorrendo transmissão de dengue ou tenha presença de *Ae.aegypti* que apresente febre, usualmente entre 2 e 7 dias, e apresente duas ou mais das seguintes manifestações: náuseas, vômitos, exantema, mialgias, cefaléia, dor retroorbital, petéquias ou prova do laço positiva e leucopenia.

Caso suspeito de Chikungunya: febre de início súbito e artralgia ou artrite intensa com inicio agudo, não explicado por outras condições, que resida ou tenha viajado para áreas endêmicas ou epidêmicas até 14 dias antes do início dos sintomas, ou que tenha vínculo epidemiológico com um caso importado confirmado.

Dados Gerais

1 Tipo de Notificação — 2 - Individual

2 Agravo/doença **1- DENGUE 2- CHIKUNGUNYA** — Código (CID10) A 90 A 92

3 Data da Notificação

4 UF

5 Município de Notificação — Código (IBGE)

6 Unidade de Saúde (ou outra fonte notificadora) — Código

7 Data dos Primeiros Sintomas

Notificação Individual

8 Nome do Paciente

9 Data de Nascimento

10 (ou) Idade — 1 - Hora 2 - Dia 3 - Mês 4 - Ano

11 Sexo M - Masculino F - Feminino I - Ignorado

12 Gestante — *1-1ºTrimestre 2-2ºTrimestre 3-3ºTrimestre 4- Idade gestacional Ignorada 5-Não 6- Não se aplica 9-Ignorado*

13 Raça/Cor — *1-Branca 2-Preta 3-Amarela 4-Parda 5-Indígena 9- Ignorado*

14 Escolaridade — 0-Analfabeto 1-1ª a 4ª série incompleta do EF (antigo primário ou 1º grau) 2-4ª série completa do EF (antigo primário ou 1º grau) 3-5ª à 8ª série incompleta do EF (antigo ginásio ou 1º grau) 4-Ensino fundamental completo (antigo ginásio ou 1º grau) 5-Ensino médio incompleto (antigo colegial ou 2º grau) 6-Ensino médio completo (antigo colegial ou 2º grau) 7-Educação superior incompleta 8-Educação superior completa 9-Ignorado 10- Não se aplica

15 Número do Cartão SUS

16 Nome da mãe

Dados de Residência

17 UF

18 Município de Residência — Código (IBGE)

19 Distrito

20 Bairro

21 Logradouro (rua, avenida,...) — Código

22 Número

23 Complemento (apto., casa, ...)

24 Geo campo 1

25 Geo campo 2

26 Ponto de Referência

27 CEP

28 (DDD) Telefone

29 Zona — 1 - Urbana 2 - Rural 3 - Periurbana 9 - Ignorado

30 País (se residente fora do Brasil)

Dados clínicos e laboratoriais

Inv.

31 Data da Investigação

32 Ocupação

Dados clínicos

33 **Sinais clínicos** **1-Sim 2- Não**

Febre — Cefaleia — Vômito — Dor nas costas — Artrite — Petéquias — Prova do laço positiva

Mialgia — Exantema — Náuseas — Conjuntivite — Artralgia intensa — Leucopenia — Dor retroorbital

34 **Doenças pré-existentes** **1-Sim 2- Não**

Diabetes — Hepatopatias — Hipertensão arterial — Doenças auto-imunes

Doencas hematolóaicas — Doenca renal crônica — Doença ácido-péptica

Dados laboratoriais

Sorologia (IgM) Chikungunya

35 Data da Coleta da 1ª Amostra (S1)

36 Data da Coleta da 2ª Amostra (S2)

Exame PRNT

37 Data da Coleta

38 Resultado — S1 S2 PRNT — 1 - Reagente 2 - Não Reagente 3 - Inconclusivo 4 - Não Realizado

Sorologia (IgM) Dengue

39 Data da Coleta

40 Resultado — 1- Positivo 2- Negativo 3- Inconclusivo 4 - Não realizado

Exame NS1

41 Data da Coleta

42 Resultado — 1- Positivo 2- Negativo 3- Inconclusivo 4 - Não realizado

43 **Isolamento** Data da Coleta

44 Resultado — 1 - Positivo 2 - Negativo 3 - Inconclusivo 4 - Não Realizado

45 **RT-PCR** Data da Coleta

46 Resultado — 1 - Positivo 2 - Negativo 3 - Inconclusivo 4 - Não Realizado

47 Sorotipo — 1- DENV 1 2- DENV 2 3- DENV 3 4 - DENV 4

48 **Histopatologia** — 1- Compatível 2-Incompatível 3- Inconclusivo 4 - Não realizado

49 **Imunohistoquímica** — 1- Positivo 2- Negativo 3- Inconclusivo 4 - Não realizado

Supplementary discussion

Here we summarize results on transmission chain reconstruction from synthetic data. Supplementary Figure 4 shows the performance of the reconstruction model for different values of Q. The average generation time was estimated with consistently high accuracy and small variability across Q. The average transmission distance was accurately estimate for Q>=12.5km and underestimated for lower values. Over 80% of transmission links were correctly identified by the model in at least one of the reconstructed chains for Q>=10km and at least 60% for smaller values of Q. The average proportion of infection sources correctly identified in each reconstructed chain was between 40% and 50% for Q >= 12.5km, with a decrease to 20% at Q=4km. About 50% of imported cases were correctly identified by the model, as long as Q remained above 15km; below this value, the ability of the model to discriminate imported cases deteriorated rapidly, with a sensitivity of about 20% at 4km; nonetheless, the model was highly specific in the identification of imported cases for all values of Q.

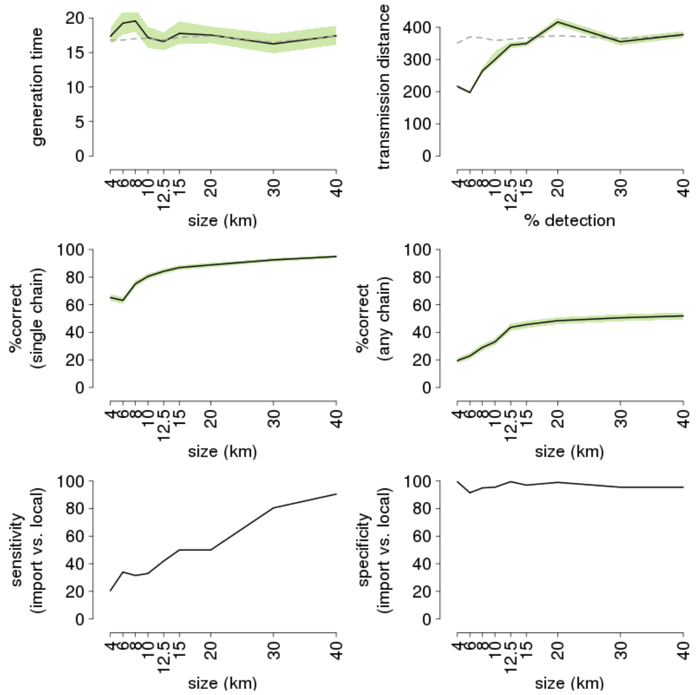

Supplementary Figure 4. Performance of the model on synthetic dataset for different sizes (Q) of the simulated study area (mean: solid black lines; 95% CI: light green areas). Dashed grey lines in the top row panels represent actual mean value in the synthetic data sets.

Supplementary Figure 5 shows a generally good agreement between the distribution of cluster sizes in synthetic datasets and in model-reconstructed transmission chains; however, the model tends to overestimate cluster sizes by merging independent clusters for Q<15km, a direct consequence of the limited sensitivity in identifying imported cases.
We further tested the model performance on this dataset by assuming different levels of underdetection of dengue cases: starting from the baseline case of Q=20km, we sampled a fraction *d* (between 10 and 90%) of the synthetic dataset. Note, however, that performances were evaluated by simulating a different epidemic for each value of the assumed notification rate *d*. The value of Q=20km was chosen in order to mimic the spatiotemporal density of cases in a given year in Porto Alegre, which has a surface of about 500 km^2. In the undersampled datasets, cases which were locally transmitted in the full dataset but whose infector was not sampled were we re-labeled as imported (since the infector would not be traceable). We then recalibrated the model on undersampled datasets and evaluated the

reconstructed transmission chains (Supplementary Figure 6). The model was able to identify the generation time with good accuracy at all underreporting levels, and the transmission distances for detection rates as low as 50%; for lower values, the transmission distance was overestimated. The proportion of sources of infection correctly identified in at least one chain decreased about linearly from over 85% in the case of perfect detection to just above 40% when *d*=10%. The proportion of sources of infection identified by each chain was consistently around 40%. The specificity with which imported cases were identified remained close to 100%, showing that the model very rarely mislabels locally transmitted cases as imported.

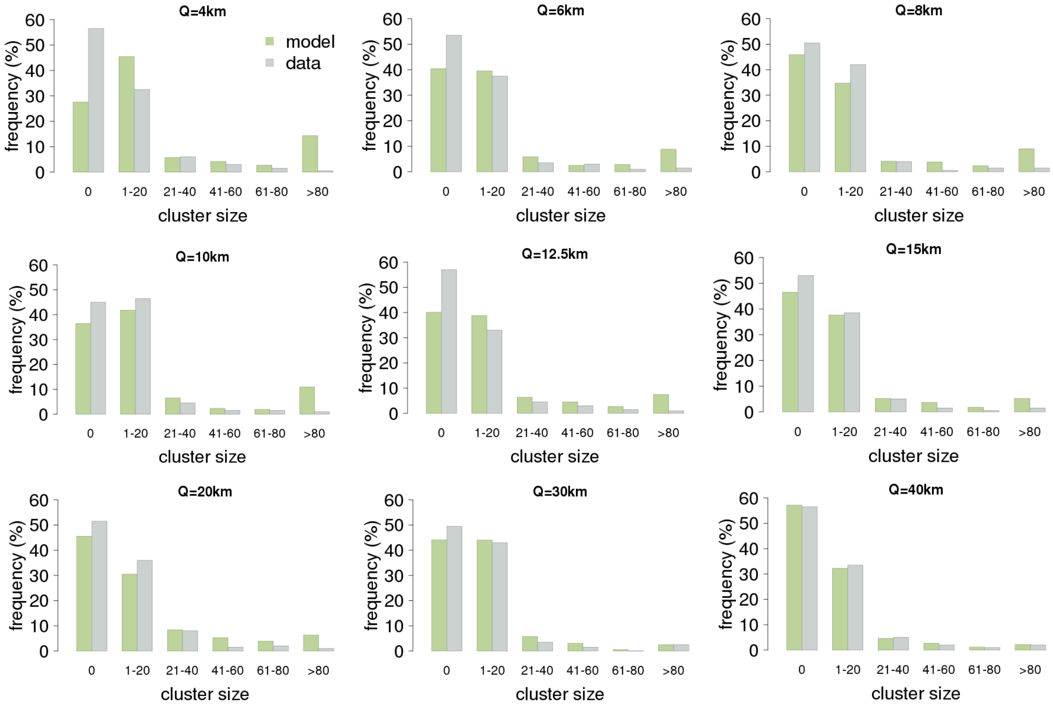

Supplementary Figure 5. Distribution of cluster sizes in synthetic datasets and as reconstructed by the model.

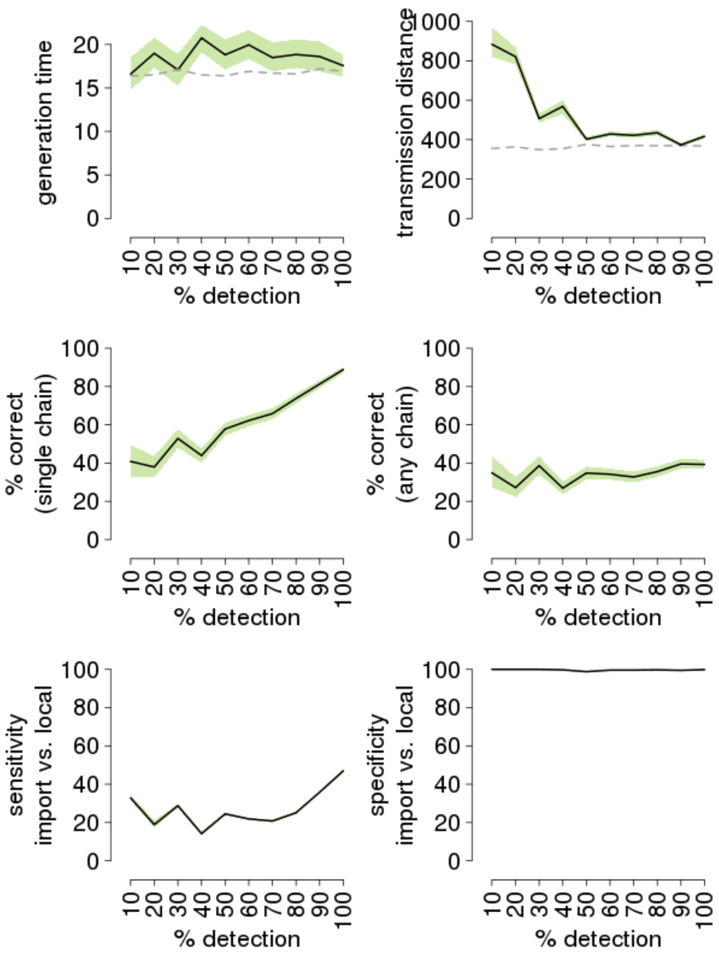

Supplementary Figure 6. Performance of the model on synthetic dataset for different detection rates and Q=20km (mean: solid black lines; 95% CI: light green areas). Dashed grey lines in the top row panels represent actual mean value in the synthetic data sets.

In Supplementary Figure 7, we show a comparison between the instantaneous reproductive number R_t, computed from the renewal equation using the gamma-distribution of the generation time obtained from the synthetic model, and the effective reproductive number R_e, estimated by the transmission chain reconstruction model for selected values of Q. There is a good agreement between the two measures, although deviations may be large in the first days of simulation because of the small number of transmission events.

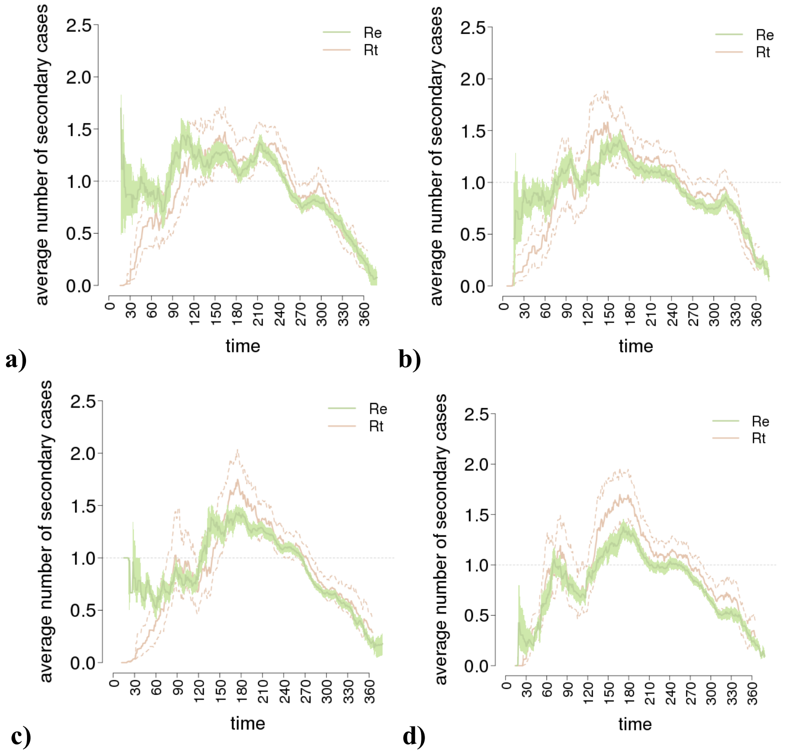

Supplementary Figure 7. Comparison of R_t and R_e for selected values of Q: a) 8km; b) 12.5km; c) 20km; d) 40km. Shaded areas and dashed lines indicate the 95% confidence interval.

Here we summarize additional results on reconstructed chains from Porto Alegre. The model was able to label correctly a large majority of locally transmitted cases in Porto Alegre: only a median of 20 (95% CI: 9-29) of the 454 known autochthonous cases were mislabeled as imported, corresponding to a median accuracy of 95.6% (95%CI: 94.0%-97.3%) (Supplementary Figure 8a; note that the number of confirmed local transmissions was very low in 2014 and 2015, so the percentages are highly variable, as shown by broad confidence intervals).

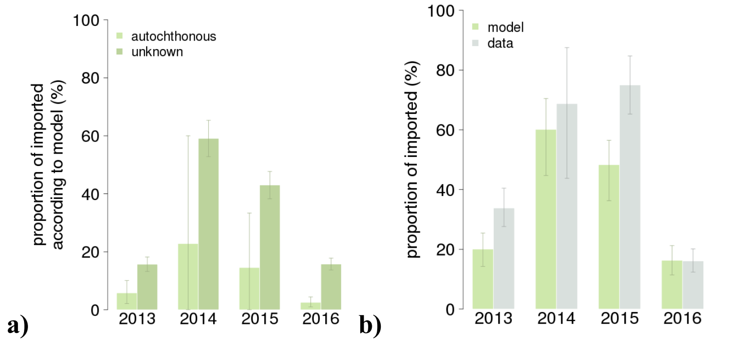

Supplementary Figure 8. a) Proportion of cases known as autochthonous labeled as imported in the model-reconstructed transmission chains (light green); proportion of cases with unknown origin labeled as imported in the model-reconstructed transmission chains (dark green). **b)** Proportion of all cases labeled as imported by the model (light green) and proportion of confirmed cases identified as imported by epidemiological investigations (grey).

For the 2767 cases with unknown classification, the label of imported case was assigned with a more than five-fold frequency (23.7% over the four years), similar to the frequency of importations in cases with known origin (29.7%, Supplementary Figure 8b).

In Supplementary Figure 9, we report the distribution of cluster durations in reconstructed transmission chains. In years with higher transmission (2013 and 2016), a higher number of clusters remained active for more than two months since the corresponding index case, while in 2014 and 2015 a significant proportion of clusters faded out within a month. The median cluster durations for the four years were, respectively, 46, 29, 33 and 55 days.

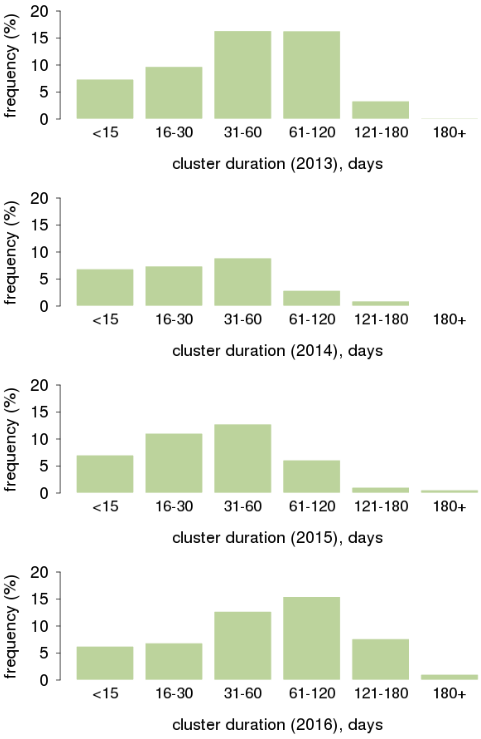

Supplementary Figure 9. Distribution of cluster duration by epidemic year (excluding importations not resulting in secondary transmission).

A recent, large-scale study using molecular epidemiological data on dengue cases from Thailand [6] showed a strong linear relationship between the number of transmission chains circulating in a given area and the area's population and population density. We found the same relationship by considering the number of transmission chains circulating in a given neighborhood and its demographic characteristics. The correlations are robust both in the case of the full dataset (Supplementary Figure 10) and with confirmed cases only (Supplementary Figure 11).

Salje et al. also found that the proportion of cases belonging to the same transmission chain decreases exponentially with distance [6]. This result is reproduced by our model, both when considering the main analysis and with confirmed dengue cases only (Supplementary Figure 12). Since the rate of decrease depends on the amount of competing transmission chains within a given area, the model predicts this decrease to be slower in the analysis with confirmed cases only.

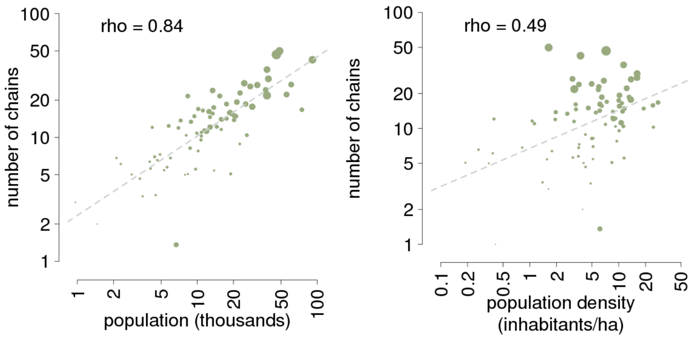

Supplementary Figure 10. Correlations (measured by Spearman's rank correlation coefficient rho) between the number of circulating chains and the total population and population density over the 81 neighborhoods included in the main analysis. Scale is log-log.

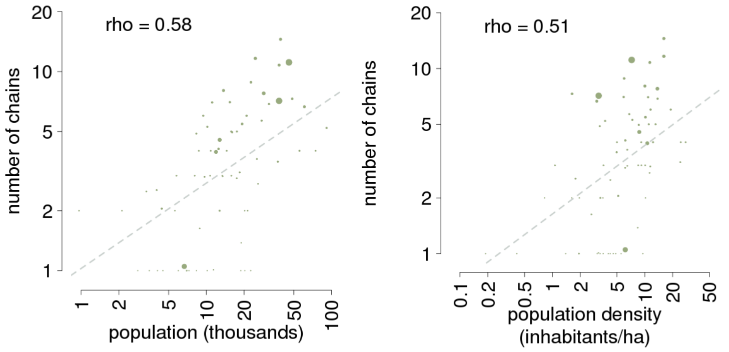

Supplementary Figure 11. Correlations (measured by Spearman's rank correlation coefficient rho) between the number of circulating chains and the total population and population density over the 70 neighborhoods included in the analysis with confirmed dengue cases. Scale is log-log.

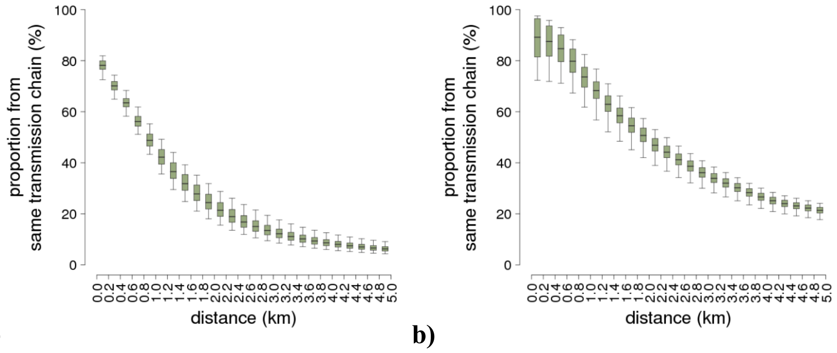

a) **b)**

Supplementary Figure 12. Decrease in the number of cases belonging to the same transmission chain over distance, using reconstructed chains from (a) suspected and confirmed dengue cases and (b) confirmed cases only.

Here we summarize results for Porto Alegre obtained by analyzing confirmed cases only. Epidemiological investigations were not able to confirm a large majority (>80%) of suspected dengue cases. It is therefore possible that some of the clinical cases included in the main analysis were due to different diseases that were co-circulating in the same years (although with a much lower intensity: for example, in the four years, there were only 34 confirmed cases of chikungunya and 29 of Zika virus). Given potential diagnostic uncertainties, we performed a complete re-analysis using only the subset of confirmed cases, representing less than 20% of the complete dataset. Despite the large undersampling, we obtained remarkably consistent results (reported below) with respect to the main analysis.

The generation time was estimated at 17.7 days (95%CI: 17.3-18.1 days). The model identified large waves of imported cases for the first months of 2013, 2015 and 2016, followed by a much more modest amount of secondary transmission, with peaks of R_e always below the epidemic threshold (Supplementary Figure 13). The low ratio of local to imported cases is a consequence of the model's difficulty in identifying a suitable infector for many cases, due to the massive undersampling in the "confirmed" dataset.

The distribution of cluster sizes was similar in the confirmed dataset with respect to the main analysis (Supplementary Figure 14), although clusters were generally smaller. The proportion of imported cases with no secondary transmission is once again higher in 2014 and 2015 (85-90%) compared to 2013 and 2016 (70-80%) and only a few clusters involved more than 10 individuals. As in the main analysis, also in this case a few larger clusters were responsible for the large majority of all secondary cases in 2013 and 2016.

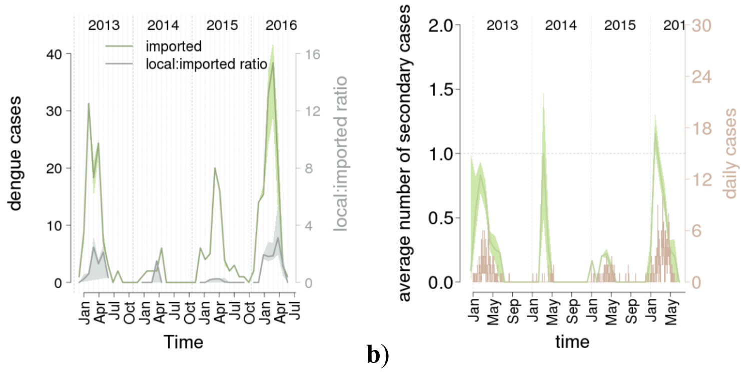

a) b)

Supplementary Figure 13. a) time series of confirmed dengue cases, classified according to model-reconstructed transmission chains. **b)** effective reproductive number R_e over time, compared with daily confirmed cases.

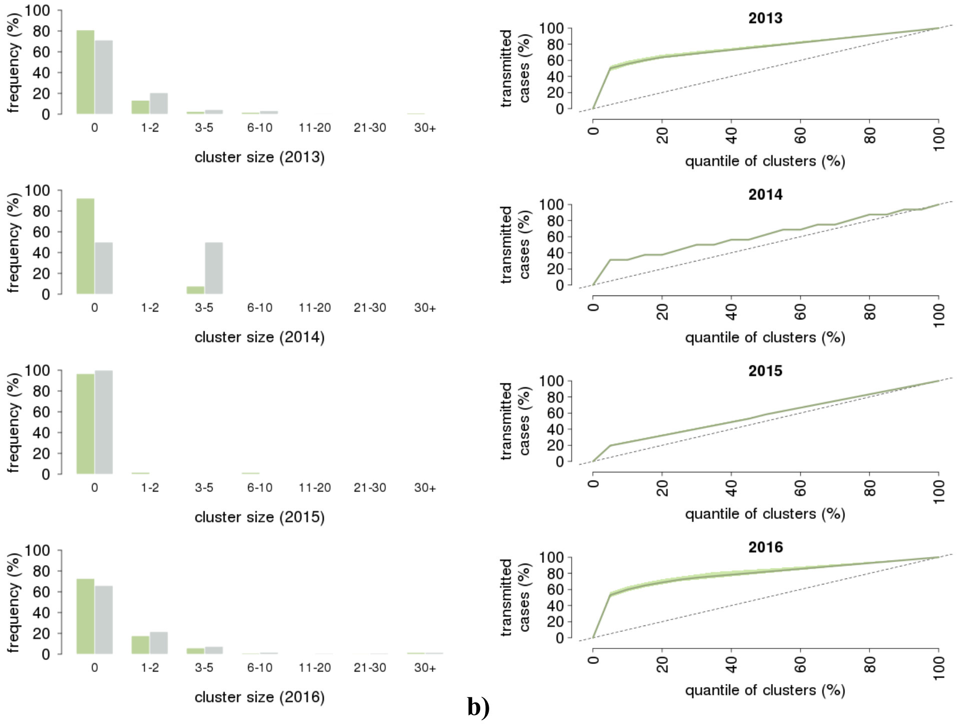

a) b)

Supplementary Figure 14. Size of transmission clusters reconstructed using the subset of confirmed dengue cases. **a)** distribution of cluster size by year; green: all clusters, grey: clusters with index case imported during the peak transmission months (February and March); **b)** cumulative number of transmitted cases by quantile of clusters

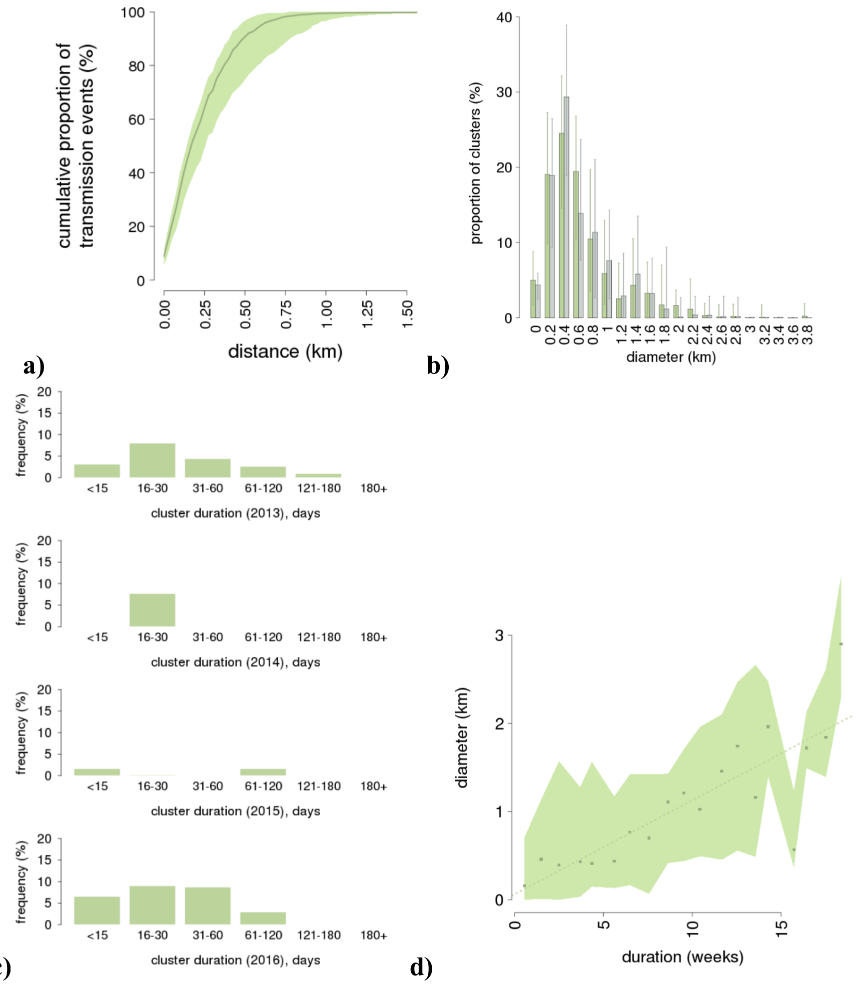

Supplementary Figure 15. Focal transmission of confirmed dengue cases: **a**) estimated cumulative proportion of transmission events as a function of distance; **b)** distribution of the cluster diameter; green: all clusters, grey: clusters with index case imported during the peak transmission months (February and March); **c)** distribution of cluster duration by year; **d)** relation between cluster diameter and duration; points: average diameter; shaded area: 95% credible interval; dashed lines: linear regression on points. Green: all clusters, orange: clusters with index case imported during the peak transmission months (February and March).

The average transmission distance was estimated at 223m (182-341m) and less than 5% of infections were transmitted beyond a distance of 1km (Supplementary Figure 15a). 85% of clusters were localized within 1km, with only 0.4% extending beyond 3km 1km (Supplementary Figure 15b). Their median duration was 26 and 29 days in 2013 and 2016 respectively (only 1 and 3 clusters of secondary confirmed cases were detected in 2014 and 2015) 1km (Supplementary Figure 15c). The linear relationship between cluster duration and cluster diameter held, but the average time required for a cluster to reach a diameter of 1km was slightly higher than in the main analysis (about 9 weeks instead of 7) (Supplementary Figure 15d).

The re-analysis on confirmed cases also shows a robust correlation between the neighborhood-specific transmission intensity and the corresponding mosquito infestation index (Supplementary Figure 16). For every doubling of the infestation index, the model estimates an increase in the transmission intensity by 77% (95%CI: 6-197%), i.e. in a similar range compared to the main analysis.

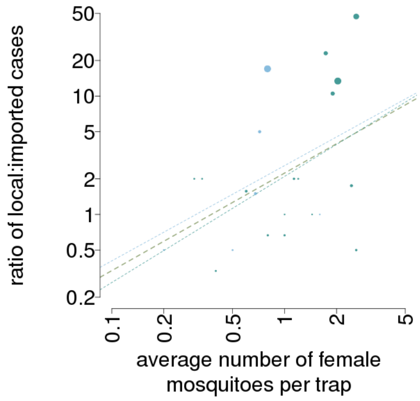

Supplementary Figure 16. Relationship between transmission intensity, represented by the ratio of local to imported cases, and the neighborhood's mosquito infestation level (log-log scale). Circles: neighborhoods for which the information on mosquito abundance was available (size is proportional to the total number of cases); Light blue: 2013; green: 2016. Dashed gray lines: linear regression model on the log-transformed variables (p-value = 0.042).

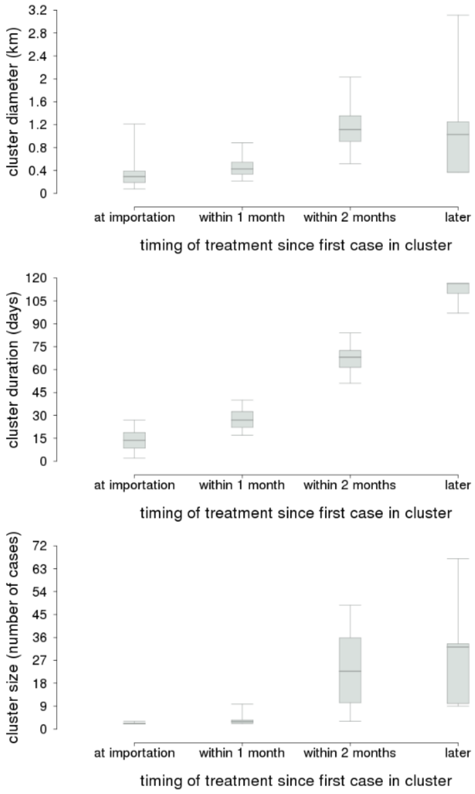

Supplementary Figure 17. Cluster characteristics with respect to timeliness of interventions. Center: mean; bounds of box: interquartile range; whiskers: 95%CI.

Supplementary Figure 17 confirms the association between cluster characteristics (diameter, duration and size) and the timeliness of interventions, although the low number of clusters in the reduced dataset prevents a meaningful disaggregation with respect to treatment intensity. The robustness of model results when sampled on the small subset of confirmed cases is somehow surprising, in light of model results on undersampled synthetic datasets. However, this can be explained by the non-random distribution of confirmed cases within transmission chains. Confirmed cases are represented in 17.5% (CI: 15.1-19.4%) of clusters reconstructed from the full dataset. By comparison, a random sample of cases with equal size (N=646) would be represented, on average, in 30.7% (27.3-34.7%) of reconstructed clusters. In addition,

confirmed cases represent, overall, 52.3% (42.1-63.6%) of the total of cases in clusters in which they appear. If confirmed cases were randomly distributed across clusters, we would expect them to represent about 20% of the total. We conclude that cases were more likely to be confirmed when they were at relatively short temporal and spatial distances from other confirmed cases, i.e. within the same cluster. In this way, confirmed cases maintain partially the spatiotemporal structure of dengue transmission of the full dataset, thereby allowing the reconstruction of key features of the transmission dynamics.

Here we summarize results obtained by analyzing under-sampled datasets from Porto Alegre. To demonstrate that the spatiotemporal structure of dengue transmission is conserved in data characterized by large underreporing rates, we re-run our analyses after discarding a fraction of randomly chosen notified cases. Supplementary Figure 18 shows that the estimated cumulative probability distribution function of transmission distances is substantially similar to the main analysis when discarding 40% (Supplementary Figure 18a) of notification data. A larger average transmission distance was estimated when discarding 80% (Supplementary Figure 18b) of notification data.

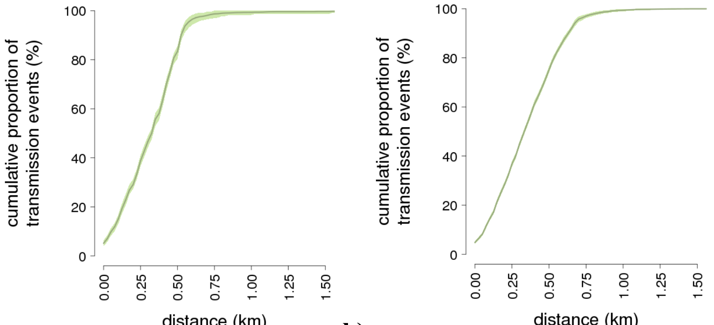

a) b)

Supplementary Figure 18. Estimated cumulative proportion of transmission events as a function of distance after randomly discarding a subset of notification data. **a)** 40% discarded cases **b)** 80% discarded cases.

Supplementary Figure 19 shows the corresponding results for cluster diffusion. In this case, the linear growth of the cluster diameter over time is qualitatively reproduced in both scenarios. The diffusion speed is also quantitatively unchanged in the scenario where 40% of notifications are discarded, but it tends to be underestimated in the scenario with 80% underreporting. Supplementary Figure 20-22 report further results for these two scenarios. All in all, these results show that results are robust when discarding up to 40% of the notification data.

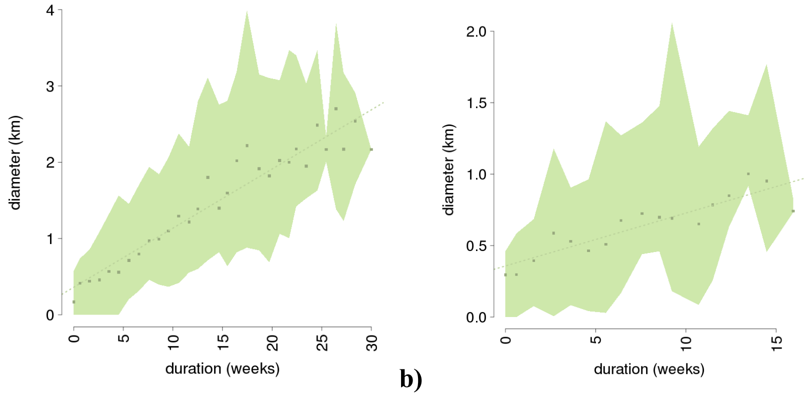

a) b)

Supplementary Figure 19. Relation between cluster diameter and duration estimated after randomly discarding a subset of notification data. **a)** 40% discarded cases **b)** 80% discarded cases. Points: average cluster diameter; shaded area: 95% credible interval; dashed lines: linear regression on points.

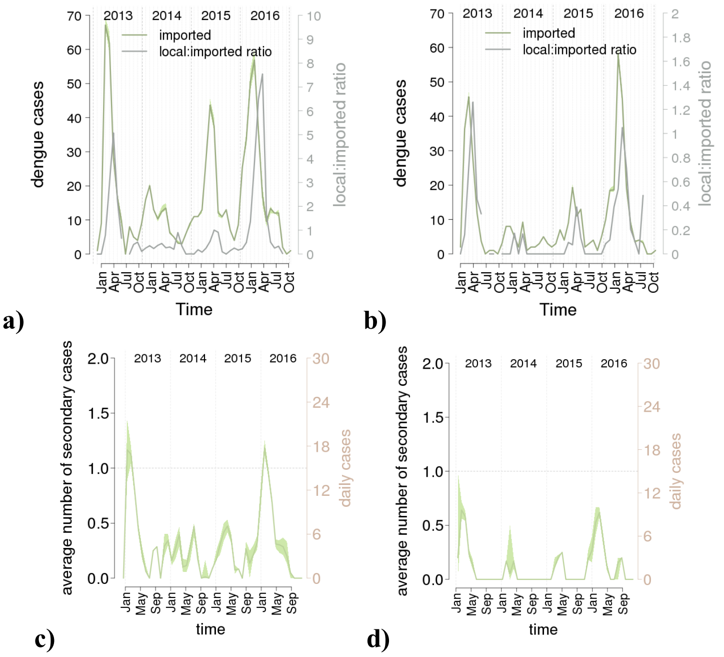

Supplementary Figure 20. a, b) time series of confirmed dengue cases, classified according to model-reconstructed transmission chains. **c, d)** effective reproductive number R_e over time. **a, c)** 40% discarded cases; **b, d)** 80% discarded cases.

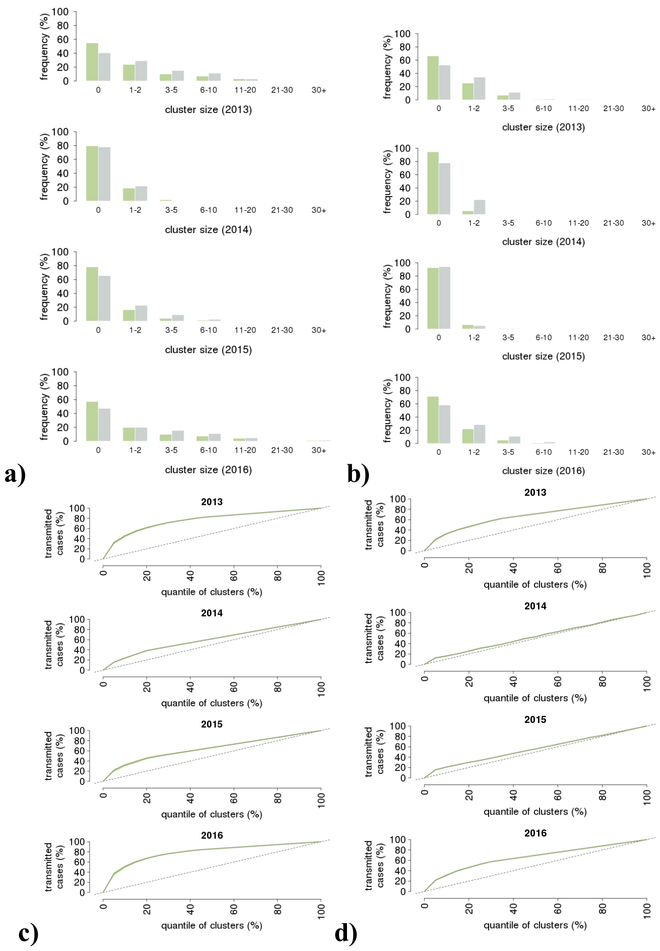

Supplementary Figure 21. Size of transmission clusters reconstructed using the subset of confirmed dengue cases. **a, b)** distribution of cluster size by year; **c, d)** cumulative number of transmitted cases by quantile of clusters. **a, c)** 40% discarded cases; **b, d)** 80% discarded cases.

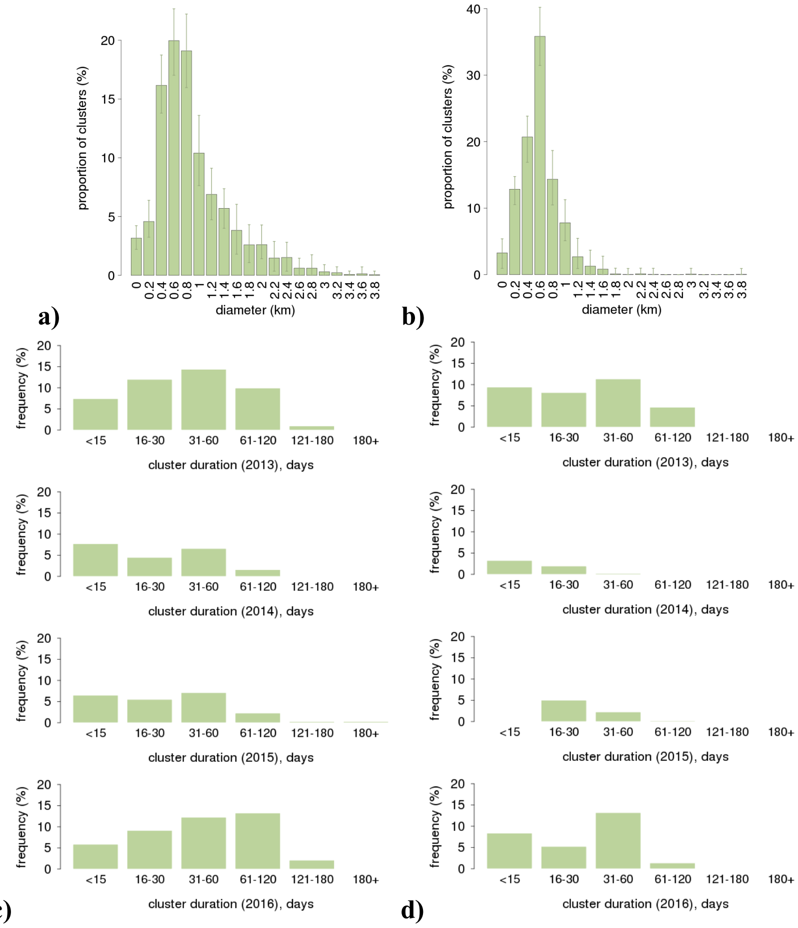

Supplementary Figure 22. a, b) distribution of the cluster diameter; **c, d)** distribution of cluster duration by year. **a, c)** 40% discarded cases; **b, d)** 80% discarded cases.

Here we summarize results for Porto Alegre obtained by using a geographic kernel based on the radiation model. To assess the role of long-distance urban commuting on dengue transmission within a city, we evaluated the model performance after substituting the geographic kernel based on negative exponential distances with a model representing human mobility. The radiation model [7] is a parsimonious and accurate way of describing human mobility and assumes that the probability of commuting from location i to location j depends on the respective populations, p_i and p_j, and on the total population p_{ij} encompassed by a circle with radius equal to the distance d_{ij} between i and j and centered on i (excluding p_i and p_j), according to the following equation:

$$K(i,j) = \frac{p_i p_j}{(p_i + p_{ij})(p_i + p_j + p_{ij})}$$

We used population data from Worldpop [8] at a spatial resolution of 1km, overlaid to official shapefiles for the administrative boundaries of Porto Alegre, and we assigned each dengue case to the nearest cell to compute the kernel values. We then recalibrated the model using the same MCMC procedure adopted for the main analysis. We compared the model performance to that of the exponential kernel using the Akaike Information Criterion [9] and Bayesian Information Criterion scores [10], in order to penalize the additional parameter η of the exponential kernel. Both the AIC and the BIC were significantly worse for the radiation model (see Supplementary Figure 23), suggesting that local transmission explains the spatiotemporal patterns of dengue transmission in Porto Alegre better than human mobility at the urban scale. Supplementary Figures 24-28 below report the main results obtained with the

radiation model. Briefly, a much larger mean transmission distance was estimated (1174m, 95%CI: 1119-1244), cluster diameter expanded up to 10km, thus covering large parts of the city (Supplementary Figure 26). Finally, the diffusion speed was estimated at roughly 2km month^{-1}. The relationship between mosquito abundance and ratio between imported and locally transmitted cases was similar to that found in the main analysis (Supplementary Figure 27). The same holds for results concerning the impact of treatment (Supplementary Figure 28).

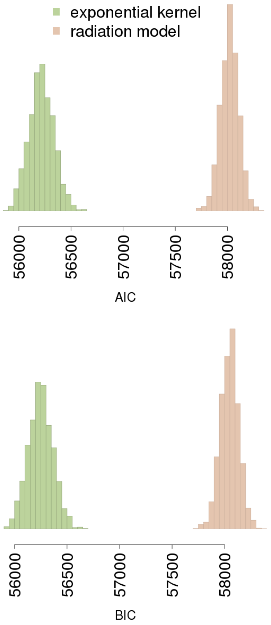

Supplementary Figure 23. Distribution of AIC and BIC scores for reconstructed transmission chains using the exponential kernel and the radiation model.

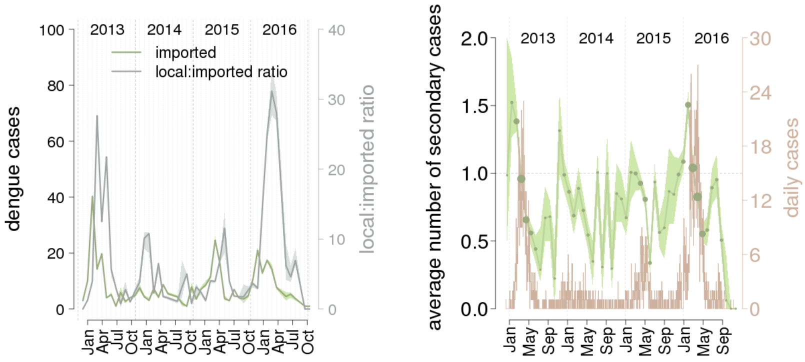

a) b)

Supplementary Figure 24. a) time series of confirmed dengue cases, classified according to model-reconstructed transmission chains using a radiation kernel. **b)** effective reproductive number R_e over time, compared with daily confirmed cases.

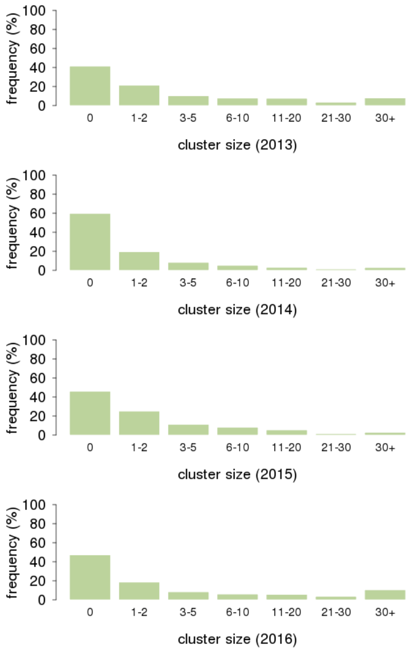

Supplementary Figure 25. Distribution of cluster size by year, reconstructed using a radiation kernel.

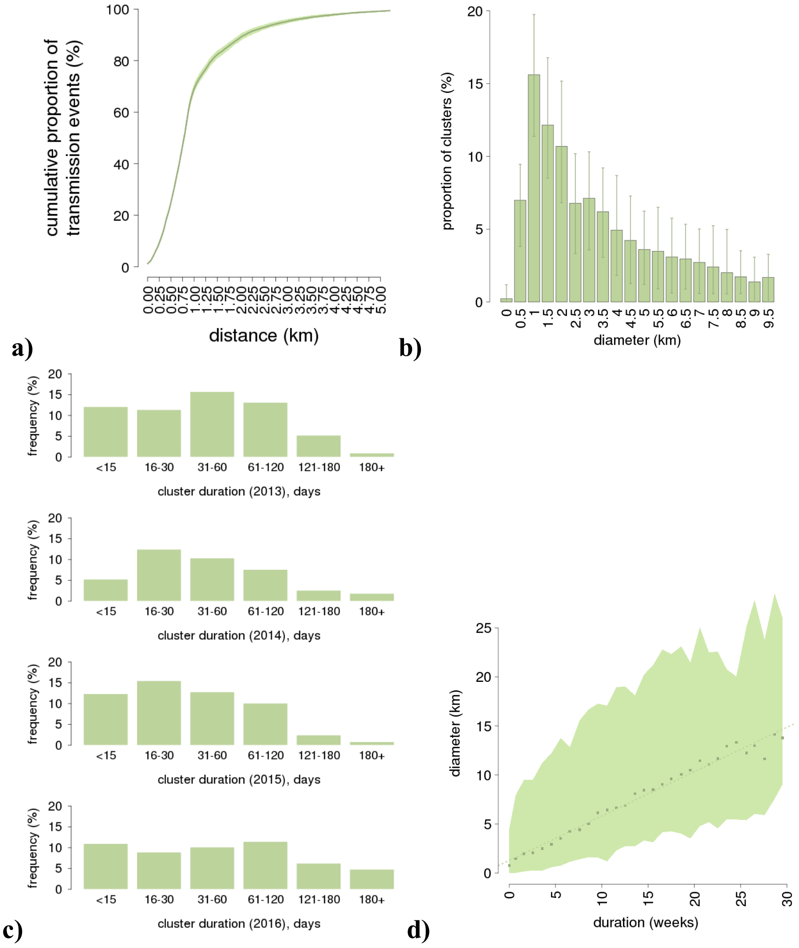

Supplementary Figure 26. Focal transmission of confirmed dengue cases reconstructed using a radiation model: **a**) estimated cumulative proportion of transmission events as a function of distance; **b)** distribution of the cluster diameter; **c)** distribution of cluster duration by year; **d)** relation between cluster diameter and duration; points: average diameter; shaded area: 95% credible interval; dashed lines: linear regression on points.

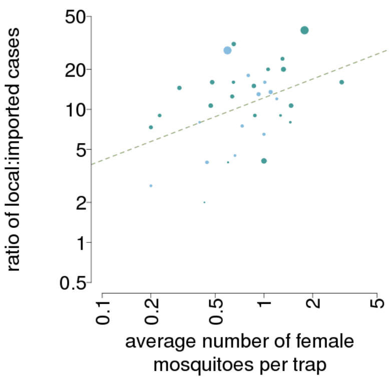

Supplementary Figure 27. Relationship between transmission intensity, represented by the ratio of local to imported cases, and the neighborhood's mosquito infestation level (log-log scale), computed from the radiation model. Circles: neighborhoods for which the information on mosquito abundance was available (size is proportional to the total number of cases); Light blue: 2013; green: 2016. Dashed gray lines: linear regression model on the log-transformed variables (p-value = 0.0001).

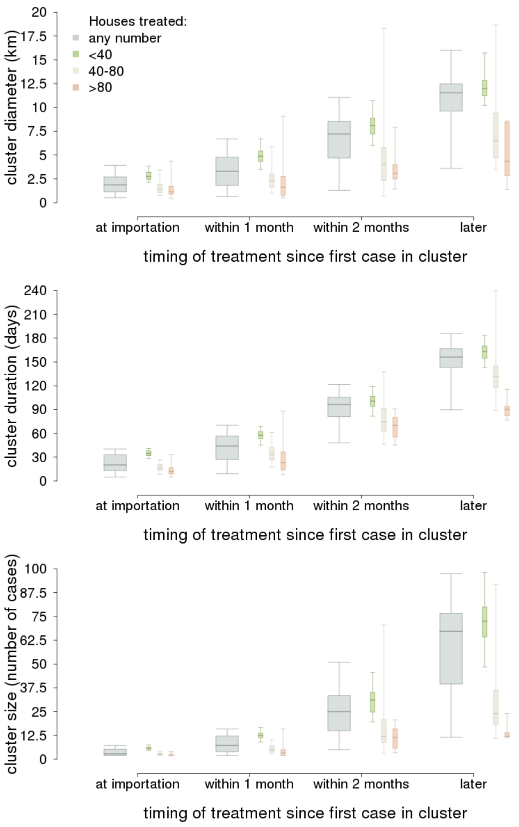

Supplementary Figure 28. Cluster characteristics with respect to timeliness and intensity of interventions. Center: mean; bounds of box: interquartile range; whiskers: 95%CI.

Supplementary References

1. Lau MS, Dalziel BD, Funk S, McClelland A, Tiffany A, Riley S, Metcalf CJ, Grenfell BT. Spatial and temporal dynamics of superspreading events in the 2014-2015 West Africa Ebola epidemic. Proc Natl Acad Sci U S A. 2017 Feb 28;114(9):2337-2342. doi: 10.1073/pnas.1614595114.
2. Gibson GJ, Renshaw E. Estimating parameters in stochastic compartmental models using Markov chain methods. Math Med Biol. 1998; 15(1):19–40.
3. Salje H, Lessler J, Paul KK, Azman AS, Rahman MW, Rahman M, Cummings D, Gurley ES, Cauchemez S. How social structures, space, and behaviors shape the spread of infectious diseases using chikungunya as a case study. Proceedings of the National Academy of Sciences. 2016 Nov 22;113(47):13420-5.
4. Chan M, Johansson MA. The incubation periods of dengue viruses. PloS one 2012; 7(11): e50972.
5. Svensson Å. A note on generation times in epidemic models. Mathematical biosciences. 2007 Jul 1;208(1):300-11.
6. Salje H, Lessler J, Maljkovic Berry I, Melendrez MC, Endy T, Kalayanarooj S, A-Nuegoonpipat A, Chanama S, Sangkijporn S, Klungthong C, Thaisomboonsuk B, Nisalak A, Gibbons RV, Iamsirithaworn S, Macareo LR, Yoon IK, Sangarsang A, Jarman RG, Cummings DA. Dengue diversity across spatial and temporal scales: Local structure and the effect of host population size. Science. 2017;355(6331):1302-1306. doi: 10.1126/science.aaj9384.
7. Simini F, González MC, Maritan A, Barabási AL. A universal model for mobility and migration patterns. Nature. 2012 Apr;484(7392):96.
8. Sorichetta A, Hornby GM, Stevens FR, Gaughan AE, Linard C, Tatem AJ. High-resolution gridded population datasets for Latin America and the Caribbean in 2010, 2015, and 2020. Scientific data. 2015 Sep 1;2:150045.
9. H. Akaike. An information criterion (AIC). Math. Sci. 14(153):5-9 (1976).
10. Schwarz G. Estimating the dimension of a model. The annals of statistics. 1978;6(2):461-4.
